# HTLV‐1 infected T cells cause bone loss via small extracellular vesicles

**DOI:** 10.1002/jev2.12516

**Published:** 2024-10-10

**Authors:** Nitin Kumar Pokhrel, Amanda R. Panfil, Haniya Habib, Shamreethaa Seeniraj, Ancy Joseph, Daniel Rauch, Linda Cox, Robert Sprung, Petra Erdmann Gilmore, Qiang Zhang, Robert Reid Townsend, Lianbo Yu, Ayse Selen Yilmaz, Rajeev Aurora, William Park, Lee Ratner, Katherine N. Weilbaecher, Deborah J. Veis

**Affiliations:** ^1^ Division of Bone & Mineral Diseases, Musculoskeletal Research Center Washington University School of Medicine Saint Louis Missouri USA; ^2^ Center for Retrovirus Research, Department of Veterinary Biosciences The Ohio State University Columbus Ohio USA; ^3^ Division Molecular Oncology Washington University School of Medicine Saint Louis Missouri USA; ^4^ Division of Endocrinology Washington University School of Medicine Saint Louis Missouri USA; ^5^ College of Public Health The Ohio State University Columbus Ohio USA; ^6^ Department of Biomedical Informatics, Bioinformatics Shared Resource, Comprehensive Cancer Center The Ohio State University Columbus Ohio USA; ^7^ Department of Molecular Microbiology and Immunology, School of Medicine Saint Louis University Saint Louis Missouri USA; ^8^ Shriners Hospitals for Children St. Louis Missouri USA; ^9^ Department of Pathology and Immunology Washington University School of Medicine Saint Louis Missouri USA

**Keywords:** adult T cell leukaemia, extracellular vesicles, osteoclast, osteolysis, proteomics

## Abstract

Adult T cell leukaemia (ATL), caused by infection with human T‐ lymphotropic virus type 1 (HTLV‐1), is often complicated by hypercalcemia and osteolytic lesions. Therefore, we studied the communication between patient‐derived ATL cells (ATL‐PDX) and HTLV‐1 immortalized CD4+ T cell lines (HTLV/T) with osteoclasts and their effects on bone mass in mice. Intratibial inoculation of some HTLV/T leads to a profound local decrease in bone mass similar to marrow‐replacing ATL‐PDX, despite the fact that few HTLV/T cells persisted in the bone. To study the direct effect of HTLV/T and ATL‐PDX on osteoclasts, supernatants were added to murine and human osteoclast precursors. ATL‐PDX supernatants from hypercalcemic patients promoted the formation of mature osteoclasts, while those from HTLV/T were variably stimulatory, but had largely consistent effects between human and murine cultures. Interestingly, this osteoclastic activity did not correlate with expression of osteoclastogenic cytokine receptor activator of nuclear factor kappa‐B ligand (RANKL), suggesting an alternative mechanism. HTLV/T and ATL‐PDX produce small extracellular vesicles (sEV), known to facilitate HTLV‐1 infection. We hypothesized that these sEV also mediate bone loss by targeting osteoclasts. We isolated sEV from both HTLV/T and ATL‐PDX, and found they carried most of the activity found in supernatants. In contrast, sEV from uninfected activated T cells had little effect. Analysis of sEV (both active and inactive) by mass spectrometry and electron microscopy confirmed absence of RANKL and intact virus. Viral proteins Tax and Env were only present in sEV from the active, osteoclast‐stimulatory group, along with increased representation of proteins involved in osteoclastogenesis and bone resorption. sEV from osteoclast‐active HTLV/T injected over mouse calvaria in the presence of low‐dose RANKL caused more osteolysis than osteoclast‐inactive sEV or RANKL alone. Thus, HTLV‐1 infection of T cells can cause release of sEV with strong osteolytic potential, providing a mechanism beyond RANKL production that modifies the bone microenvironment, even in the absence of overt leukaemia.

## 1 INTRODUCTION

Human T‐lymphotropic virus type‐1 (HTLV‐1) is a retrovirus with an estimated 15–20 million carriers worldwide. HTLV‐1 spreads through contact with bodily fluids containing infected cells via virological synapses, but poorly as a free virus (Lairmore et al., 2011). Adult T cell leukaemia (ATL), an aggressive, treatment‐refractory malignancy, occurs in 2%–5% of patients infected with the virus, often decades after initial infection (Qayyum & Choi, 2014). Clinical presentation often includes hypercalcemia, a life‐threatening complication, due to release of the mineral from bone (Kiyokawa et al., 1987; Prager et al., 1994). Previous studies of ATL have shown expression of soluble factors such as receptor activator of nuclear factor kappa‐B ligand (RANKL), Macrophage Inflammatory Protein‐ Alpha (MIP1α), and tumour necrosis factor (TNF) (Kohart et al., 2019; Nosaka et al., 2002; Okada et al., 2004; Parrula et al., 2009; Prager et al., 1994; Shu et al., 2010; Xiang et al., 2019) which directly enhance formation of osteoclasts, the myeloid‐lineage polykaryons responsible for both physiologic and pathologic bone resorption. ATL cells also can produce parathyroid hormone‐related protein (PTHrP), a growth factor associated with many forms of hypercalcemia of malignancy, that exerts its effects indirectly, via the osteolineage (Grunbaum & Kremer, 2022; Kohart et al., 2019; Shu et al., 2010). Bone is also a rich source of matrix‐stored growth factors such as transforming growth factor β (TGF‐β) and insulin‐like growth factor 1 (IGF1) (Rieunier et al., 2019; Trivedi et al., 2021) which can interact with tumour cells in bone, affecting tumour growth and dormancy. In addition, recent studies have shown that tumour cells in non‐skeletal primary sites communicate with the bone microenvironment, preparing it to be receptive to local tumour cell dissemination and growth, that is formation of a premetastatic niche (Foster et al., 2022; Taipaleenmäki, 2023). It has been shown, using mouse models, that alterations to the bone microenvironment are also important for hematologic malignancies (Giannakoulas et al., 2021; Kode et al., 2014). However, factors favouring transition to malignancy following chronic viral infection, such as with HTLV‐1, are largely unknown. Here we investigate communication from HTLV‐1‐infected T cells to osteoclasts, which may represent a first step.

A number of model systems have been used to study the osteolysis associated with ATL, employing tumour cell lines, HTLV‐1 infection of humanized mice, and transgenic mice (Niewiesk, 2016). We have previously published that established ATL cell lines have multiple phenotypes (osteolytic, osteoblastic and mixed) when implanted locally into bone, and their expression of soluble osteoclastogenic factors is not uniform (Kohart et al., 2019). HTLV‐1 infection of humanized mice or implantation of patient‐derived ATL cells (ATL‐PDX) in mice cause systemic bone loss, but these models have rapidly progressive leukaemia or polyclonal lymphoproliferation associated with systemic inflammation, confounding the analysis of bone pathophysiology and evaluation of early events in the transition to malignancy (Xiang et al., 2019). Furthermore, human anti‐RANKL antibody provides incomplete protection from bone loss in the infection model (Xiang et al., 2019). We have also studied the bone phenotype of transgenic mice expressing the HTLV‐1 oncogenes Tax and Hbz under the granzyme B (GzmB) promoter (Esser et al., 2017; Gao et al., 2005). Tax, a coactivator of cAMP response element‐binding protein (CREB) binding proteins, activates many viral and cellular genes and is capable of inducing inflammation as well as transformation of multiple cell types. Tax^GzmB^ transgenic mice develop subcutaneous tumours composed of cells that are fully transformed and transplantable but have characteristics of NK cells rather than T cells. Hbz, a helix‐basic loop zipper protein, is expressed at late stages in ATL and regulates proliferation as well as expression of inflammatory factors including RANKL (Xiang et al., 2019). Hbz^GzmB^ transgenic mice develop lymphoproliferation of both CD4 and CD8 positive cells, although ATL cells are almost always CD4 positive. In both transgenic lines, mice develop osteolysis and hypercalcemia, with a long latency. Thus, although useful, each of these mouse models have key differences in pathophysiology compared to human ATL.

In addition to releasing the many soluble factors previously studied in the context of osteolysis, HTLV‐1 infected cells, like uninfected cells, release small extracellular vesicles (sEV), membrane‐bound vesicles enriched in multiple biological cargos including proteins, metabolites, miRNAs and mRNAs (Urabe et al., 2021). Initially considered mere waste‐carrying vehicles, sEV are now recognized as important mediators of intercellular communication in homeostasis and pathologic conditions including cancer (Becker et al., 2016). An early study of sEV released by HTLV‐1 infected cells demonstrated transfer of the oncogene Tax (Jaworski et al., 2014). These sEV increase viral spread and disease progression during HTLV‐1 infection by promoting cell to cell contact (Pinto et al., 2019; Pinto et al., 2021). More generally, sEV from tumour cells are believed to provide signalling cues for initiation of metastasis, immune evasion and preparation of the pre‐metastatic niche (Becker et al., 2016; Urabe et al., 2021). Studies with cell lines have demonstrated direct osteoclastogenic effects of breast and prostate tumour‐derived sEV (Urabe et al., 2023; Wu et al., 2021), and indirect effects via enhanced RANKL production by osteolineage cells in multiple myeloma (Pitari et al., 2015). sEV from ATL cell lines and PDX were shown to modulate bone marrow stromal cell proliferation and gene expression, suggesting that they could modify the bone microenvironment (El‐Saghir et al., 2016). However, to date there are no published studies examining effects of sEV on bone in vivo, in the context of ATL or HTLV‐1 infection.

In this study, we utilized a panel of HTLV‐1 infected T cell lines to study their interactions with osteoclasts in culture as well as in mouse models, in comparison with ATL‐PDX. We found that sEV from both HTLV‐1‐infected T cell lines and ATL‐PDX can promote in vitro osteoclastogenesis and in vivo bone resorption. In addition, these cell lines lead to consistent in vivo osteolysis in mice independent of RANKL/OPG expression.

## 2 METHODS

### 2.1 Animals

6‐week‐old NOD‐*Prkdc^em26Cd52^Il2rg^em26Cd22^*/NjuCrl (NCG) mice (Charles River, Wilmington, MA) were used for in vivo experiments with cell and sEV implantation. The numbers of male and female mice were matched in all the experimental groups. Mice were kept with ad libitum access to fresh chow and water in a pathogen‐free temperature‐controlled barrier facility. Mice were monitored daily by staff of the Division of Comparative Medicine. 6−8 week‐old immune‐competent C57Bl/6 female mice were used for bone marrow macrophage (mBMM) isolation. All the animal experimental protocols were approved by Washington University School of Medicine's Animal Studies Committee (approvals 19‐1059 and 22‐0339).

### 2.2 Generation of HTLV/T cells

HTLV‐1 immortalized CD4+ T cell lines (HTLV/T) were created by co‐culturing lethally irradiated HTLV‐1 producer cells with freshly isolated human peripheral blood mononuclear cells (PBMCs). After a period of 10–14 weeks, HTLV‐1‐immortalized CD4+ T‐cells were established and were maintained in RPMI with 20% FBS, 1% pen/strep and 20 U/mL human IL‐2. After establishment of cell lines, they were validated for surface expression of CD3 and CD4 by flow cytometry (Maksimova et al., 2023; Maksimova et al., 2022).

### 2.3 Maintenance of ATL/PDX

PBMCs from human ATL patients were expanded in culture in RPMI with 20% FBS in the presence of human IL‐2 (20 U/mL) in the beginning. They were subsequently cultured in decreasing IL‐2 concentrations until they became IL‐2 independent, over 1–3 weeks as clonal expansion progressed.

### 2.4 mBMM isolation and osteoclast differentiation

Bone marrow was harvested from dissected femurs and tibiae of 2‐month‐old C57Bl/6 mice via centrifugation at 12,000 × *g* for 2 min. Resuspended marrow was passed through a 40 µM filter, and cells cultured in alpha MEM (Sigma, M0894) containing 10% FBS (Gibco), 100 IU/mL penicillin/streptomycin, and 1:10 dilution of CMG 14–12 cell supernatant containing an equivalent of 100 ng/mL macrophage‐colony stimulating factor (M‐CSF) for 5–7 days. The resulting expanded mBMM were plated 10,000 cells/well in 96‐well plates. Purified GST‐RANKL (Novack et al., 2003) at 10 ng/mL was added to alpha MEM containing 10% FBS, 100 IU/mL penicillin/streptomycin, and a 1:50 dilution of CMG14‐12 cell supernatant (Takeshita et al., 2000). The medium was changed every alternate day. HTLV/T supernatants (10% volume:volume) or unconditioned culture media (vehicle) were added as indicated. TRAP staining was performed after fixation with 4% paraformaldehyde (Polysciences, Warrington, PA) and 0.1% Triton X‐100 in PBS according to the manufacturer's instructions (Sigma, 387A). The plates were left to air dry and imaged using a light microscope (Olympus BX41, Tokyo, Japan).

### 2.5 PBMC isolation and osteoclast differentiation

PBMCs were isolated by density gradient centrifugation of the blood from anonymous healthy donors. PBMCs were cultured in the presence of 10 ng/mL RANKL and 1:50 dilution of CMG14‐12 cell supernatant for 5–7 days. HTLV/T supernatants were used for mBMM cultures. TRAP staining was performed as for murine cultures.

### 2.6 Resorption assay

Expanded BMM (5 × 10^5^ cells) were seeded on 6‐well plates and treated with 50 ng/mL of RANKL for 48 h for the generation of pre‐osteoclasts. These were lifted and plated on bovine bone slices with or without 10% HTLV/T supernatant together with 10 ng/mL of RANKL and 1:50 dilution of CMG14‐12 cell supernatant. After 5 days, bone slices were incubated in 0.5 NaOH for 30 s, then rinsed in PBS. Cells were removed using a cotton swab and bone slices were incubated with 20 µg/mL peroxidase‐conjugated wheat germ agglutinin in PBS (Sigma, 61767) for 30 min. Bone slices were washed three times in PBS and incubated with DAB Chromogen kit (#BDB20004H, Biocare Medical, Biocare Medical, Pacheco, CA) at 37°C for 30 min. Air‐dried slices were imaged by a light microscope as above.

### 2.7 In vivo micro‐computed tomography

The right tibiae of mice were scanned by micro‐computed tomography (micro‐CT) in vivo (Viva‐CT 40, Scanco, Brüttisellen, Switzerland) at 10.5 µm resolution (70 kVp, 114 mA, 8 W, 100 ms integration time) at 2‐ or 4‐week intervals. Cancellous bone parameters were measured in a 1 mm region distal to the end of the tibial growth plate (Bouxsein et al., 2010).

### 2.8 Ex vivo micro‐CT

The calvaria of mice were scanned by micro‐CT ex vivo at 7 µm resolution (70 kVp, 57uA, 4 W, 700 ms integration time). Calvarial bone volume fraction (BV/TV) encompassed 200 slices at each region of interest.

### 2.9 Genomic DNA quantitative PCR

Genomic DNA was prepared from flushed marrow of cell line‐injected tibiae using DNeasy Blood & Tissue Kit (#69504, Qiagen, Hilden, Germany,). A non‐injected mouse bone marrow was used as a negative control. A standard curve was created by spiking a known number of human cells in increasing percentage into a fixed number of mouse bone marrow cells. Quantitative PCR program was run on ABI QuantStudio 3 with iTaq Universal SYBR Green Supermix (#1725121, Bio‐Rad, Hercules, CA,) with human *Il6* and mouse *B2m* primers, using species‐specific regions (Table S1). Each reaction proceeded at 50°C for 2 min, 95°C for 10 min, and then 40 cycles of 95°C for 15 s and 60°C for 1 min. The relative amount of human DNA was calculated as 2^^‐(hIL6 CT ‐mB2M CT)^. The percentage of human cells was calculated by plotting values against the standard curve.

### 2.10 sEV isolation

One million HTLV/T cells were cultured in RPMI supplemented with 20% FBS, 1% Penicillin/Streptomycin and 20 IU human IL‐2 (Roche Diagnostics, Mannheim, Germany) for 5 days. FBS was depleted of large vesicles by centrifugation of heat inactivated serum at 2000 × *g* for 15 min at room temperature, followed by another centrifugation of supernatant at 17.5K × *g* (35,000 × *g*) for 15 min at 4°C. Vesicles were isolated using either of two commercial exosome isolation reagents (Total Exosome Isolation Reagent #4478359, Thermo Fisher Scientific; ExoQuick Exosome Isolation #EXOTC‐10A, SBI, Palo Alto, CA) using the manufacturers’ protocols. The precipitated sEV were resuspended in PBS and stored at −80°C. No significant differences were observed in the activity of sEV between these kits. Isolated sEV were analysed for size and distribution using NanoSight NS300 (Malvern Panalytical, Worcestershire, UK). All relevant data from our experiments have been submitted to the EV‐TRACK knowledgebase (EV‐TRACK ID: EV240142) (EV‐TRACK Consortium et al., 2017).

### 2.11 sEV uptake assay

mBMM were grown on coverslips. sEV were labelled with PKH 26 (MINI 26, Sigma, St. Louis, MO) following the manufacturer's protocol. The cells were treated with labelled sEV for 4 h. Images were captured using Leica DMi8 automated inverted microscope equipped with ACS APO 20×/0.60 Lens (Leica Microsystems, Wetzler, Germany).

### 2.12 RNA isolation and reverse transcription quantitative real‐time PCR

After cell lysis with TRIzol (Life Technologies), solution was subjected to phenol:chloroform extraction followed by centrifugation at 12,000 × *g*, and the aqueous layer was harvested. An equal volume of 70% ethanol was added, and the remaining RNA isolation was done using the NucleoSpin RNA II Kit (Clontech Laboratories, Palo Alto, CA; 740955.50). One microgram RNA was transferred into the cDNA Ecodry Premix Kit prior to the quantitative PCR program being run on ABI QuantStudio 3 with iTaq Universal SYBR Green Supermix (Bio‐Rad, Hercules, CA, 1725121). Each reaction proceeded at 50°C for 2 min, 95°C for 10 min, and then 40 cycles of 95°C for 15 s and 60°C for 1 min. Relative expression was calculated as 2^^‐(target CT ‐control CT)^. The primer sequences used are listed in Table S1.

### 2.13 Intratibial injection of cells

Mice were kept under anaesthesia with isoflurane and the right knee joint was exposed and sterilized. A 27G needle was inserted through the knee joint to make a hole, 2.5 × 10^6^ cells in 10 µL PBS were injected, and the skin incision was sutured. Buprenorphine SR was given as a pain reliever.

### 2.14 Calvarial injection of sEV

NCG mice (6 weeks of age) were anaesthetized with isoflurane and injected subcutaneously over the calvarium once per day. For the first 2 days, all mice received 50 µg RANKL, and next 3 days 25 µg RANKL along with either PBS or sEV (9 × 10^7^) from HTLV/T C7 or C8. Calvaria were harvested for micro‐CT 3 days after the last treatment.

### 2.15 Transmission electron microscopy

sEV were fixed with 1% glutaraldehyde (Ted Pella Inc., Redding CA) and allowed to absorb onto freshly glow‐discharged formvar/carbon‐coated copper grids (200 mesh, Ted Pella Inc.) for 10 min. Grids were then washed two times in dH_2_O and stained with 1% aqueous uranyl acetate (Ted Pella Inc) for 1 min. Excess liquid was gently wicked off and grids were allowed to air dry. Samples were viewed on a JEOL 1200EX transmission electron microscope (JEOL USA, Peabody, MA) equipped with an AMT 8‐megapixel digital camera (Advanced Microscopy Techniques, Woburn, MA).

### 2.16 Small RNA sequencing

RNA was isolated from precipitated sEV using Sera Mir (Exosome RNA Column Purification Kit #RA808A‐1, SBI). Total RNA integrity was determined using Agilent Bioanalyzer or 4200 Tapestation. Library preparation was performed with 1ug of total RNA with a Bioanalyzer RIN score greater than 8.0. Full library preparation was performed using the TruSeq Small RNA kit (Illumina, San Diego, CA) per manufacturer's protocol. Fragments were sequenced on an Illumina NextSeq‐550 using single reads extending 75 bases. Basecalls and demultiplexing were performed with Illumina's bcl2fastq software with a maximum of one mismatch in the indexing read. RNA‐seq reads were then aligned to the Ensembl release 101 primary assembly with STAR version 2.7.9a1. Gene counts were derived from the number of uniquely aligned unambiguous reads by Subread: feature Count version 2.0.32. Isoform expression of known Ensembl transcripts was quantified with Salmon version 1.5.23. Sequencing performance was assessed for the total number of aligned reads, total number of uniquely aligned reads, and features detected. The ribosomal fraction, known junction saturation, and read distribution over known gene models were quantified with RSeQC version 4.04.

### 2.17 Proteomics

sEV isolated from human HTLV/T cell cultures with Total Exosome Isolation Reagent (#4478359, Thermo Fisher Scientific) were solubilized in SDS buffer, digested, and quantified, then analysed using trapped ion mobility time‐of‐flight mass spectrometry. Comprehensive methodology and data processing are provided in the supplemental text.

### 2.18 Histology

Mouse calvaria were fixed in 10% neutral buffered formalin for 24 h followed by decalcification in 14% Free Acid EDTA (pH 7.2) for 5 days. 7µM sections were prepared and subjected to TRAP staining. Number of osteoclasts per bone perimeter (NOc/BPm) was calculated using Image J.

### 2.19 Statistical analysis

All statistics were computed using SAS software (Version 9.4, SAS Institute Inc., Gary, NC), GraphPad Prism software (Version 10.1.1, GraphPad Software, Inc., La Jolla, CA). Values of *p*  <  0.05 were considered significant and data are presented as mean  ±  SD. For two group comparisons, a student's, paired or unpaired, two‐tailed *t*‐test was used. For multiple group comparisons, a 1‐way ANOVA followed by Tukey's multiple comparisons test was performed. For repeated measure group comparisons, linear mixed‐effects models were used followed by Tukey's method for multiplicity adjustment. Specific statistical tests and sample sizes are indicated in the respective figure legends.

## 3 RESULTS

### 3.1 HTLV‐1 infected T cells cause bone loss in mice even in the absence of overt leukaemia

**FIGURE 1:**
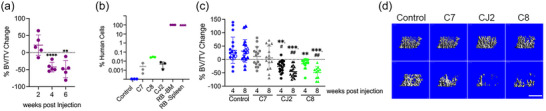
Implantation of HTLV‐1 infected T cell lines into bone causes osteolysis even in the absence of overt leukaemia. (a) NCG mice were injected intra‐tibially with 250,000 ATL‐PDX RB in the right tibia and trabecular bone volume fraction (BV/TV) was examined by viva‐CT every 2 weeks (*n* = 5). Percent change from baseline (prior to inoculation) is indicated. (b) Genomic DNA PCR for amount of human cells in bone marrow of injected tibiae after 8 weeks of injection for HTLV/T lines, together with RB bone marrow and spleen after 6 weeks of injection, and mouse bone marrow as negative control. (c) NCG mice were injected intra‐tibially with 250,000 HTLV/T lines C7 (grey), CJ2 (black) and C8 (green). Control mice were injected with PBS (open circles) or were uninjected (closed circles). BV/TV of the injected leg was determined by viva‐CT before and 4‐ and 8‐week post injection. Data represented as %BV/TV change from baseline (*n* = 11–18 mice per group). (d) Representative micro‐CT images from mice in (c). Scale bar 100µm. (a), (c); Linear mixed‐effects model with Tukey's method for multiplicity adjustment, ^**^ *p* < 0.01, ^***^ *p* < 0.001 or ^****^ *p* < 0.0001 versus control; ^#^ *p* < 0.05 or ^##^ *p* < 0.01 versus C7. HTLV/T, HTLV‐1 immortalized CD4+ T cell lines.

Previously, we reported that established ATL cell lines, directly inoculated into bone, have variable ability to cause in vivo osteolysis (Kohart et al., 2019). In contrast, intraperitoneal administration of patient‐derived cell lines (ATL‐PDX) or infection of humanized mice with HTLV‐1 leads to massive systemic bone loss, but these mice also have rapidly progressive leukaemia or lymphoproliferation (Xiang et al., 2019) making it difficult to discern whether the bone loss is directly caused by HTLV‐1 infection or is a consequence of high systemic T cell burden. To address this, we locally injected RB, an ATL‐PDX derived from a hypercalcemic patient, in one tibia of non‐humanized NCG mice and monitored bone density by viva‐CT. As expected, RB caused massive local bone loss by four weeks (Figure 1a), but these mice also developed lethal systemic leukaemia with concomitant splenomegaly and complete bone marrow and splenic replacement within 6 weeks (Figure 1b).

To develop an alternative model for studying interactions between HTLV‐1 infected T cells and bone cells, we generated nine distinct HTLV‐1 immortalized T cell lines (HTLV/T) from two different healthy donors. These HTLV/T cell lines were created by co‐culturing lethally irradiated HTLV‐1 producer cells with freshly isolated human PBMCs, which after 12–14 weeks established HTLV‐1 infected, IL‐2 dependent immortalized T cell lines. We chose three HTLV/T cell lines (C7, CJ2, C8) which grew well in vitro, and injected these into non‐humanized 6‐week‐old NCG mice, intra‐tibially. Using quantitative PCR on genomic DNA, we found that very small percentages of HTLV/T survived in mice, compared to the complete marrow replacement with ATL‐PDX RB (Figure 1b). Using larger cohorts of mice, we assessed trabecular bone volume fraction (BV/TV) by viva‐CT at 0, 4 and 8 weeks after inoculation. As expected, bone density increased in control mice as they are still growing at 6 weeks of age. Of the 3 HTLV/T lines implanted, CJ2 and C8 caused a profound decrease in bone mass. In contrast, even though C7‐injected mice have a trend towards blunting of bone accrual, the change is not significantly different from control, and bone mass remained significantly higher than those inoculated with CJ2 or C8 (Figure 1c,d). This suggests that HTLV‐1 infected T cells can drive bone loss independent of leukaemia, and in fact do so even when present in very small numbers.

### 3.2 sEV from HTLV/T cell lines stimulate in vitro osteoclastogenesis

**FIGURE 2:**
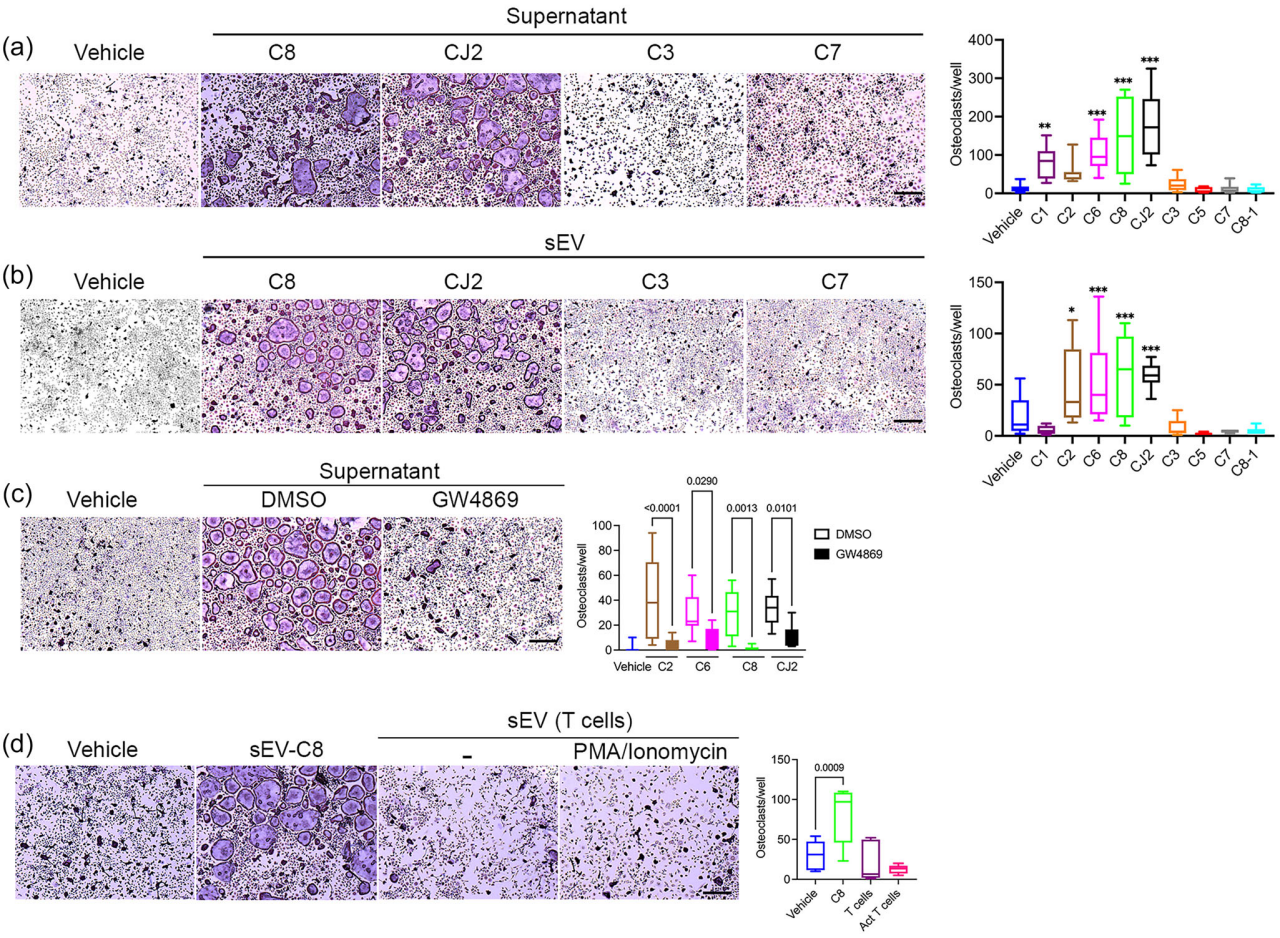
sEV from osteolytic HTLV/T cell lines stimulate in vitro osteoclastogenesis. (a) mBMM were treated with either vehicle or 10% supernatant together with low‐dose RANKL for 4 days. TRAP positive multinuclear cells were counted as osteoclasts. *n* = 9, from at least three independent experiments. (b) mBMM were treated with ∼ 6 × 10^6^ sEV isolated from culture supernatants or vehicles for 4 days. TRAP positive multinuclear cells were counted as osteoclasts. Data from three independent experiments, *n* = 9. (c) HTLV/T cells were pre‐treated with GW4869 (10 µM) or DMSO for 5 days and 10% supernatant was added during 4 days of differentiation. Data from three biological repeats, *n* = 9. (d) sEV were isolated from IL‐2 expanded human PBMC, stimulated (or not) with PMA (50 ng/mL) and ionomycin (1 nM), and added (∼6 × 10^6^ sEV) to osteoclastogenic cultures for 4 days. Results from two independent experiments, *n* = 6. Scale bars 500 µm. All statistics one‐way ANOVA, _*_ *p* < 0.05_,_^**^ *p* < 0.01, ^***^ *p* < 0.001. HTLV/T, HTLV‐1 immortalized CD4+ T cell lines; PBMC, peripheral blood mononuclear cells; RANKL, receptor activator of nuclear factor kappa‐B ligand; sEV, small extracellular vehicle.

We next tested whether osteolytic HTLV/T cell line supernatants have a direct effect on osteoclasts. To this end, we cultured mBMM with HTLV/T cell line supernatant (10%) in the presence of a low‐dose of RANKL, which is unable to form mature osteoclasts. We observed that C8 and CJ2, along with C1 and C6, had statistically significant stimulatory effect on generation of multinuclear osteoclasts, with C2 showing a trend towards positive effect, while other cell lines had little or no effect (Figure 2a). Indeed, a similar pattern of activity among supernatants was observed in human osteoclast cultures (Figure S1a,b), in which there was a significant effect on osteoclast size, greater in magnitude than the effect on their number (Figure S1c). As expected, supernatants from ATL‐PDX, derived from hypercalcemic patients, consistently stimulated multinucleated osteoclast formation from murine precursors (Figure S2a–c).

We previously showed that ATL cells express RANKL, driven by viral HBZ expression, and anti‐RANKL antibody treatment offered moderate protection from bone loss in HTLV‐1‐infected humanized mice (Xiang et al., 2019). Therefore, we quantitated the cellular mRNA level of RANKL, the RANKL antagonist OPG, and their ratio in HTLV/T clones. They expressed variable amounts of these factors and displayed no correlation to osteoclast numbers (Figure S2d). We also observed that ATL‐PDX display varying expression of RANKL and OPG (Figure S2e). This suggested that additional factors in the supernatant other than RANKL and OPG might dictate the osteolytic phenotype.

Because sEV have been shown to play a role in HTLV‐1 transmission and progression in a humanized mouse model (Pinto et al., 2021), we hypothesized that the sEV carry the osteoclast‐stimulatory activity of HTLV/T supernatants. We therefore precipitated sEV from culture supernatant using commercial kits, and confirmed their size distribution and concentration (Figure S3a). We found no difference in sEV size and concentration between osteoclast‐active and inactive groups (Figure S3b,c). Furthermore, we validated sEV preparations by performing proteomics on sEV isolated by a kit‐based precipitation method and a V‐96 peptide‐based method. The protein profile of sEV isolated by two different methods was comparable (Table S2). We also demonstrated their uptake by mBMM (Figure S3d). The sEV isolated from stimulatory supernatants similarly promoted multinucleated OC formation, whether derived from ATL‐PDX or HTLV/T lines (Figures S4a–b and 2b) in a dose‐dependent manner (Figure S4c–d), except for sEV from HTLV/T C1 supernatant. Moreover, sEV from unconditioned media, contributed by FBS, had no effect on OC differentiation (Figure S4e–f). To further strengthen the evidence for a stimulatory role of sEV rather than soluble factors in culture supernatants, we pre‐treated HTLV/T cell lines with a chemical inhibitor of EV generation (GW4869), which did not affect cell viability (Figure S5a). The quantity of sEV isolated trended lower with GW4869, due to the remaining contribution of FBS in the media (Figure S5b–e). In all 4 HTLV/T lines tested, the number of mature osteoclasts was decreased when these supernatants from GW4869‐treated cells were added to mBMM, compared to supernatants from DMSO control treated cells (Figure 2c).

HTLV‐1 infection leads to T cell activation (Kataoka et al., 2015). Since activated T cells are known to stimulate osteoclastogenesis, we sought to determine whether such activation, rather than viral infection itself, leads to production of osteoclast‐active sEV. We utilized PBMC, cultured in IL‐2 with or without stimulation by PMA and ionomycin for 2 days, and isolated sEV (Figure S6). We observed that sEV from activated T cell cultures failed to impact osteoclasts (Figure 2d). These results indicate that the osteoclastogenic activity of sEV is specific for those derived from T cells infected by HTLV‐1.

### 3.3 HTLV‐1 infected T cells promote the maturation of osteoclasts and do not act on precursor bone marrow macrophages

**FIGURE 3:**
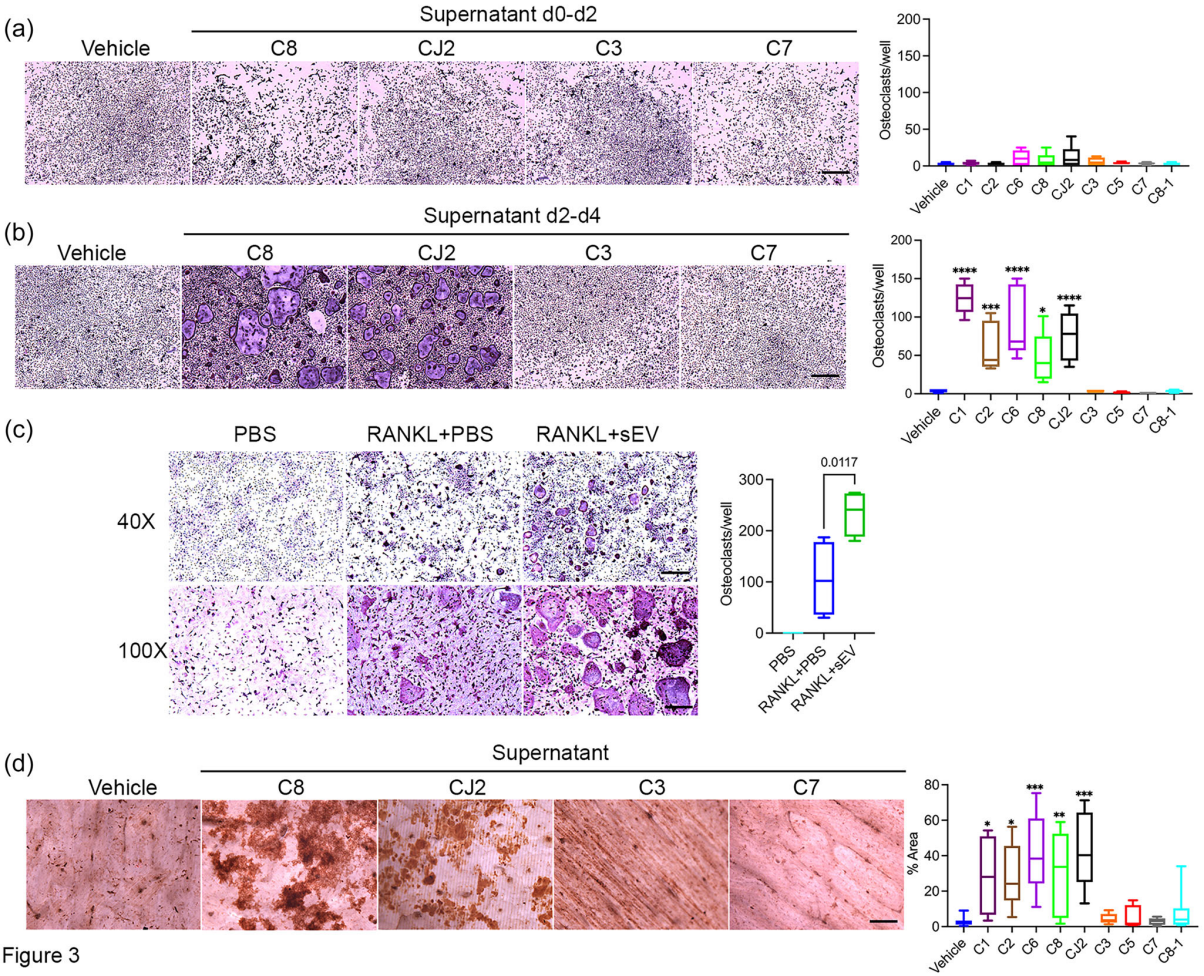
HTLV/T cells modulate maturation of osteoclasts. (a), (b) HTLV/T supernatants were added to osteoclastogenic mBMM cultures during the first 2 days (a) or last 2 days (b). Cells were fixed and TRAP stained after 4 days, and osteoclasts were counted. (c) Pre‐osteoclasts were generated by treating mBMM with 50 ng/mL of RANKL for 2 days. These were then cultured with low‐dose RANKL with or without sEV (from HTLV/T C8) for an additional 24 h. (d) Pre‐osteoclasts were lifted and replated on bone slices, treated with supernatant for 5 days, and resorption pits were stained. Resorption area was measured using ImageJ. *n* = 6–9, from 2 to 3 independent experiments. Scale bars: 500 µm—(a), (b), and (c) (upper panel); 200 µm—(c) (lower panel), (d) all statistics one‐way ANOVA, ^*^ *p* < 0.05, ^**^ *p* < 0.01_,_^***^ *p* < 0.001, ^****^ *p* < 0.0001. HTLV/T, HTLV‐1 immortalized CD4+ T cell lines; RANKL, receptor activator of nuclear factor kappa‐B ligand; sEV, small extracellular vesicles.

To determine if HTLV/T stimulated the osteoclast differentiation program, we examined osteoclast‐specific gene expression after treatment with culture supernatants. Surprisingly, we found no increase in marker levels compared to vehicle controls (Figure S7a). This prompted us to examine which stage of osteoclastogenesis was targeted by HTLV/T cells. Osteoclast differentiation can be broadly divided into two stages, generation of committed pre‐osteoclasts and their subsequent fusion to multinucleated giant cells (Veis & O'Brien, 2023). We performed a time course study where we added supernatant either for the first 2 days or last 2 days of differentiation, which indicated that HTLV/T targeted the latter maturation stage (Figure 3a,b). Similarly, pre‐osteoclasts generated in low‐dose RANKL for 2 days and then treated with sEV for an additional day formed more multinucleated osteoclasts than those treated with PBS (Figure 3c). As with supernatant‐treated cells, there was no change in expression of differentiation genes in sEV‐treated cultures (Figure S7b). To determine if the generated osteoclasts were functional, we cultured pre‐osteoclasts as above, plated them on bone slices, then added supernatant for 5 more days, and examined resorption activity. As expected, HTLV/T supernatants with high activity on multinucleation also had greater effects on resorptive potential (Figure 3d). This suggests that HTLV/T culture supernatants enhanced the fusion of pre‐osteoclasts to generate mature, bone‐resorbing cells.

### 3.4 Profiling of sEV from HTLV‐1 infected T cells identifies proteins and miRNAs that regulate osteoclastogenesis

**FIGURE 4:**
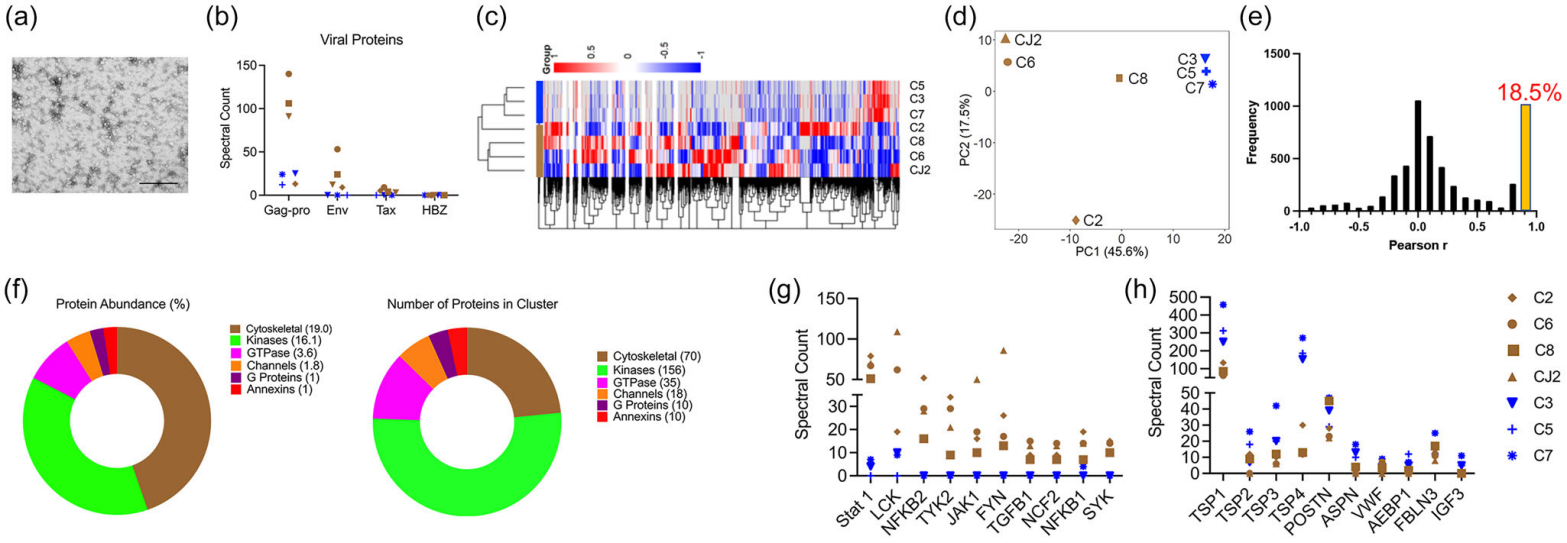
Proteomics of sEV suggests multiple possible candidates modulating osteoclastogenesis. (a) Representative transmission electron micrograph, of sEV isolated from C8. Scale bar: 100 nm. (b)–(h) LC‐MS/MS analysis was performed on proteins isolated from sEV with positive (*n* = 4, brown), and negligible (*n* = 3, blue) effect on osteoclast differentiation. (b) Analysis of viral proteins shows presence of gag‐pro in all sEV, but Env (gp62) and Tax only in sEV that stimulate osteoclasts. HBZ was not detected in any. (c)–(h) Analysis of cellular proteins, (c) heat map, (d) PCA plot, (e) Pearson's correlation, (f) gene ontogeny analysis. (g) STRING network analysis displaying enriched proteins in sEV with high osteoclastogenic effect, (h) STRING network analysis displaying enriched proteins in sEV without osteoclastogenic effect. sEV, small extracellular vesicles.

We performed transmission electron microscopic (TEM) analysis of both HTLV/T and ATL‐PDX sEV and found them to be electro‐lucent and lacking dense virion cores, confirming the absence viral particles in our sEV preparations (Figures 4a and S8a), similar to what others have demonstrated with ultracentrifugation‐based isolation of sEV from HTLV‐1 infected cells (Jaworski et al., 2014). We then performed LC‐MS/MS on osteoclast‐active (*n* = 4) and inactive (*n* = 3) HTLV/T sEV groups. Analysis confirmed the presence of sEV markers according to minimal information for studies of extracellular vesicles (MISEV) guidelines (Welsh et al., 2024) in all seven sEV preparations (Figure S8b). HTLV/T sEV carry only three HTLV‐1 viral polypeptides, of which two (Env and Tax) are only present in sEV with stimulatory effects on osteoclasts (Figure 4b). Notably, neither RANKL nor OPG was detected in any of the sEV. To gain further insight into components of the sEV, we examined protein abundance by heat map (Figure 4c) and clustering between the groups by PCA plot (Figure 4d). These results both showed separation of active versus inactive groups of sEV. Furthermore, correlation analysis revealed that among 5248 proteins carried in the cargo, 968 (18.5%) proteins were highly correlated with the osteoclastogenic phenotype (Figure 4e). Of those highly correlated proteins, gene ontology analysis showed that most of the proteins are cytoskeletal, GTPases, and kinases, which could have a role in influencing fusion and resorptive function (Figure 4f). We used STRING to understand the protein‐protein interactions and found that osteoclast differentiation and TCR signalling pathways overlapped in this analysis (Figure S9). The osteoclast differentiation proteins were enriched in the active group, (Figure 4 g) while inactive sEV had an abundance of thrombospondins (Figure 4h). Proteomics data was confirmed by examining levels of Flotillin‐1 (EV marker), Calreticulin (non‐EV marker), Env (high in osteoclast‐active sEV) and Thrombospondin 1 (high in osteoclast‐inactive sEV) by western blotting (Figure S10). We also performed miRNA sequencing on the active sEV group. As expected, these sEV were rich in miRNAs and the top miRNAs in all 4 active sEV preparations were similar (Table S3). Interestingly, of those, three miRNAs (mir155, mir21, mir92a1) were previously reported to have a positive effect on osteoclast differentiation (Hu et al., 2017; Mao et al., 2019; Yu et al., 2021). These results suggest that both the protein and miRNAs in sEV cargo have the potential to affect osteoclast maturation and function.

### 3.5 sEV from HTLV‐1 infected T cells are sufficient to cause in vivo osteolysis in non‐tumour bearing mice

**FIGURE 5:**
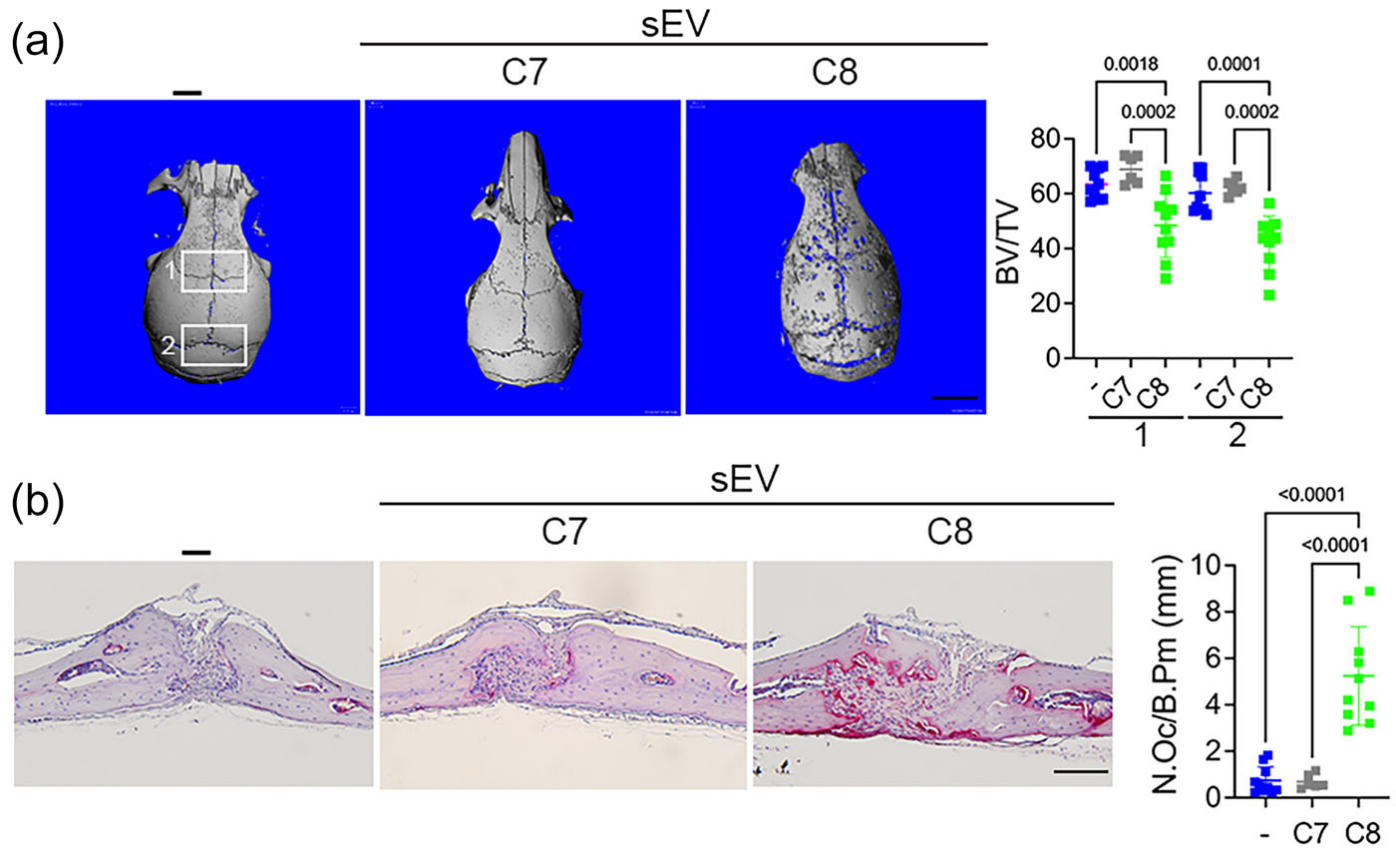
HTLV/T sEV causes bone loss in calvarial osteolysis model. Mice were injected with RANKL together with PBS or sEV (9 × 10^7^) from HTLV/T C7 or C8 subcutaneously over the calvaria. (a) Calvaria were scanned using micro‐CT and bone volume fraction (BV/TV) was determined in two regions, as indicated. Scale bar: 1 mm. (b) Calvaria were decalcified, and paraffin embedded sections were subjected to TRAP staining. NOc/BPm was counted. *n* = 6–10/group. Scale bar: 100 µm. One‐way ANOVA, *p*‐values indicated. HTLV/T, HTLV‐1 immortalized CD4+ T cell lines; NOc/BPm, osteoclast number per bone perimeter; RANKL, receptor activator of nuclear factor kappa‐B ligand; sEV, small extracellular vesicles.

We next sought to establish whether HTLV/T‐derived sEV directly induces osteolysis in vivo. As we had determined that sEV induced formation of mature osteoclasts only in RANKL‐primed cultures, we used a similar approach in a mouse osteolysis model. We injected RANKL over the calvarium for 2 days to initiate OC formation, then treated with a low‐dose of RANKL alone or with sEV from C7 (a non‐stimulatory HTLV/T line) and C8, (a stimulatory HTLV/T line), for 3 more days. Mice were euthanized 3 days after the last treatment, allowing time for osteoclasts to resorb bone. Consistent with its effect in vitro, application of C8 sEV to calvaria increased pit formation and apparent width of sutures, leading to a marked decrease in bone mass, compared to those treated with C7 sEV or RANKL alone (Figure 5a). Upon histological examination, we observed a significant increase in TRAP positive osteoclasts only upon C8 sEV treatment (Figure 5b). These results indicate that sEV released by HLTV‐1 infected cells have the ability to stimulate osteoclasts in vivo, leading to osteolysis.

## 4 DISCUSSION

To understand the role of HTLV‐1 infection in bone loss, we generated HTLV‐1 infected, immortalized T cell lines and implanted them intra‐tibially. Interestingly, although these cells persisted at low levels in the bone marrow over 8 weeks, some HTLV/T cell lines caused progressive bone loss. This osteolytic activity correlated with in vitro ability of supernatants derived from these cells to stimulate the formation of mature osteoclasts in culture. The finding is quite remarkable given that very few HTLV/T cells survive in the bone marrow, but they have a strong and sustained effect, causing a similar amount of bone loss to ATL‐PDX models which replace the normal marrow constituents. Further analysis indicated that sEV, rather than RANKL or other soluble proteins, were the primary drivers of bone loss. Consistent with their in vitro effect, isolated osteoclast active sEV injected over the calvarium caused rapid osteolysis in mice. Although activated T cells are known to stimulate osteoclastogenesis via RANKL expression, their sEV did not, indicating that the osteolytic potential of sEV was specific to HTLV‐1 infection.

Expression of sEV markers, defined by MISEV guidelines (Welsh et al., 2024), were similar between the sEV from the HTLV/T cell lines with or without effects on osteoclast differentiation. Similar to sEV from HTLV‐1‐infected cells isolated by other groups, these sEV lacked complete viral particles, and were also devoid of RANKL and OPG. Our osteoclastogenic sEV shared other features with prior studies. Jaworski et al. found that HTLV‐1‐infected T cells release sEV with viral protein Tax (Jaworski et al., 2014). Interestingly, in our study, Env and Tax were only present in sEV with osteoclast stimulatory activity. Env in particular has been found to be fusogenic (Hoshino, 2012) and may play a similar role to enhance multinucleation of osteoclasts in our cultures utilizing suboptimal doses of RANKL. Formation of very large, hyper‐multinucleated osteoclasts is characteristic in Paget's Disease, which can be associated with paramyxovirus infection (Kurihara et al., 2011). Notably, however, we have not observed sEV‐induced multinucleation beyond the levels obtained with higher, optimized doses of RANKL in the absence of sEV. Osteoclastogenic sEV were also enriched for many proteins identified in other studies to be involved in osteoclast differentiation and function (Veis & O'Brien, 2023). Additionally, the most abundant miRNAs, also found in sEV by El‐Saghir, 2016, were miRNA155 and miRNA21, both with osteoclastogenic effects (Hu et al., 2017; Mao et al., 2019; Pitari et al., 2015). Thus, the osteoclast‐active sEV bear a complex cargo with multiple components with the potential to enhance osteoclast maturation and resorptive function. It is therefore unlikely that modulation of a single component will eliminate their activity.

The ability of very small numbers of HTLV/T cells to cause bone loss in immunodeficient mice in vivo was surprising. These cells are dependent on human IL‐2 in vitro, and we did not transplant human hematopoietic stem cells (i.e. humanization) nor did we provide an exogenous source of IL‐2. It is possible that provision of IL‐2 or other T cell supportive cytokines would lead to significant lymphoproliferation in vivo. Nevertheless, this fortuitous finding of extensive osteolysis points to the presence of a potent mediator. Important to our conclusion that sEV are the primary candidates of osteoclast activation is the absence of an in vivo effect of implanted C7 HTLV/T cells, which persist at similar levels to highly osteolytic CJ2. Neither the whole supernatant nor isolated sEV from C7 are stimulatory in vitro or in vivo. Nano Sight analysis confirmed similar concentrations and size distributions of isolated sEV, and proteomic analysis showed that C7, and other inactive sEV, were enriched in some proteins compared to the active sEV, suggesting a distinct cargo rather than defective sEV production by these cell lines. We thought it was important to directly apply sEV in vivo to assess their osteolytic effects. Because the HTLV/T cells grow slowly, we were unable to inject large numbers systemically over weeks, as others have done for solid tumour‐derived sEV (Urabe et al., 2023; Wu et al., 2021). Instead, we used only three injections locally over the calvarium, and the number of sEV was 1000‐fold lower than these other studies. Whether HTLV/T‐derived sEV are particularly potent in their effects on bone will require direct comparisons with sEV from other sources. Additionally, the reciprocal role of the bone microenvironment on HTLV‐1 infected T cells, prior to and after transformation, remains to be investigated.

Interestingly, we observed that sEV from HTLV/T cells with minimal effect in osteoclastogenesis were enriched in thrombospondins, markers associated with asymptomatic carriers of HTLV‐1 (Ferreira et al., 2012). It is intriguing to speculate that a preponderance of sEV with thrombospondins might predict a lower propensity to ATL progression, compared to those with enrichment of markers such as Env found at high levels in osteolytic sEV. A previous proteomic analysis of plasma‐derived sEV from HTLV‐1 infected patients failed to detect viral proteins (Jeannin et al., 2018). Thus, isolation of sEV specifically from T cells might be needed to achieve adequate sensitivity. Significantly, more investigation of potential prognostic utility is warranted of sEV from HTLV‐1 infected patients with a range of disease types, including asymptomatic, and HTLV‐associated myelopathy, leukaemia and lymphoma.

In sum, our data shows that HTLV‐1 infection of T cells leads to production of sEV that can drive bone loss in vivo, independent of the development of systemic leukaemia. Our understanding of sEV as a modulator of osteoclastogenesis could be beneficial for providing a better understanding of bone‐tumour interactions, designing new clinical therapies, and using sEV for predicting disease progression.

## AUTHOR CONTRIBUTIONS

**Nitin Kumar Pokhrel**: Data curation (lead); formal analysis (lead); investigation (lead); methodology (lead); writing—original draft (lead); writing—review and editing (lead). **Amanda R. Panfil**: Conceptualization (supporting); methodology (supporting); writing—review and editing (supporting). **Haniya Habib**: Formal analysis (supporting). **Sham Seeniraj**: Formal analysis (supporting). **Ancy Joseph**: Investigation (supporting); methodology (supporting). **Daniel Rauch**: Formal analysis (supporting). **Linda Cox**: Investigation (supporting). **Robert Sprung**: Investigation (supporting). **Petra Erdmann Gilmore**: Investigation (supporting). **Qiang Zhang**: Formal analysis (supporting). **Robert Reid Townsend**: Conceptualization (supporting); methodology (supporting); supervision (supporting). **Lianbo Yu**: Formal analysis (supporting). **Ayse Selen Yilmaz**: Formal analysis (supporting). **Rajeev Aurora**: Formal analysis (supporting). **William Park**: Formal analysis (supporting). **Lee Ratner**: Conceptualization (supporting); formal analysis (supporting); funding acquisition (supporting); supervision (supporting); writing—review & editing (supporting). **Katherine N. Weilbaecher**: Conceptualization (supporting); formal analysis (supporting); funding acquisition (supporting); supervision (supporting); writing—review and editing (supporting). **Deborah J. Veis**: Conceptualization (lead); funding acquisition (lead); project administration (lead); supervision (lead); writing—original draft (lead); writing—review and editing (lead).

## CONFLICT OF INTEREST STATEMENT

The authors report no conflict of interest. This project was funded by the National Institutes of Health, NCI P01 CA100730, which provided direct support to most co‐authors (N.K.P., A.P., H.H., S.S., A.J., D.R., L.C., L.Y., A.Y.S., L.R., K.N.W. and D.J.V.). The cores of the Washington University Musculoskeletal Research Center were supported by NIH P30 AR074992. We also acknowledge the Molecular Microbiology Imaging Facility for TEM image acquisition. The proteomic experiments were performed at the Washington University Proteomics Shared Resource (WU‐PSR), supported in part by the Washington University Institute of Clinical and Translational Sciences (NCATS UL1 TR000448), the Mass Spectrometry Research Resource (NIGMS P41 GM103422) and the Siteman Comprehensive Cancer Center Support Grant (NCI P30 CA091842). We thank the Genome Technology Access Center at the McDonnell Genome Institute at Washington University School of Medicine for help with genomic analysis. The Center is partially supported by NCI Cancer Center Support Grant #P30 CA91842 to the Siteman Cancer Center from the National Center for Research Resources (NCRR), a component of the National Institutes of Health (NIH), and NIH Roadmap for Medical Research. This publication is solely the responsibility of the authors and does not necessarily represent the official view of NCRR or NIH.

## Supporting information



Supporting Information
