# Supplementary material for: HTLV‐1 infected T cells cause bone loss via small extracellular vesicles
Source: J Extracell Vesicles. 2024 Oct 10;13(10):e12516. doi: 10.1002/jev2.12516 (PMC11464911; doi:10.1002/jev2.12516)
Supplement: Supplementary file 1 — Supporting Information [file JEV2-13-e12516-s001.docx]

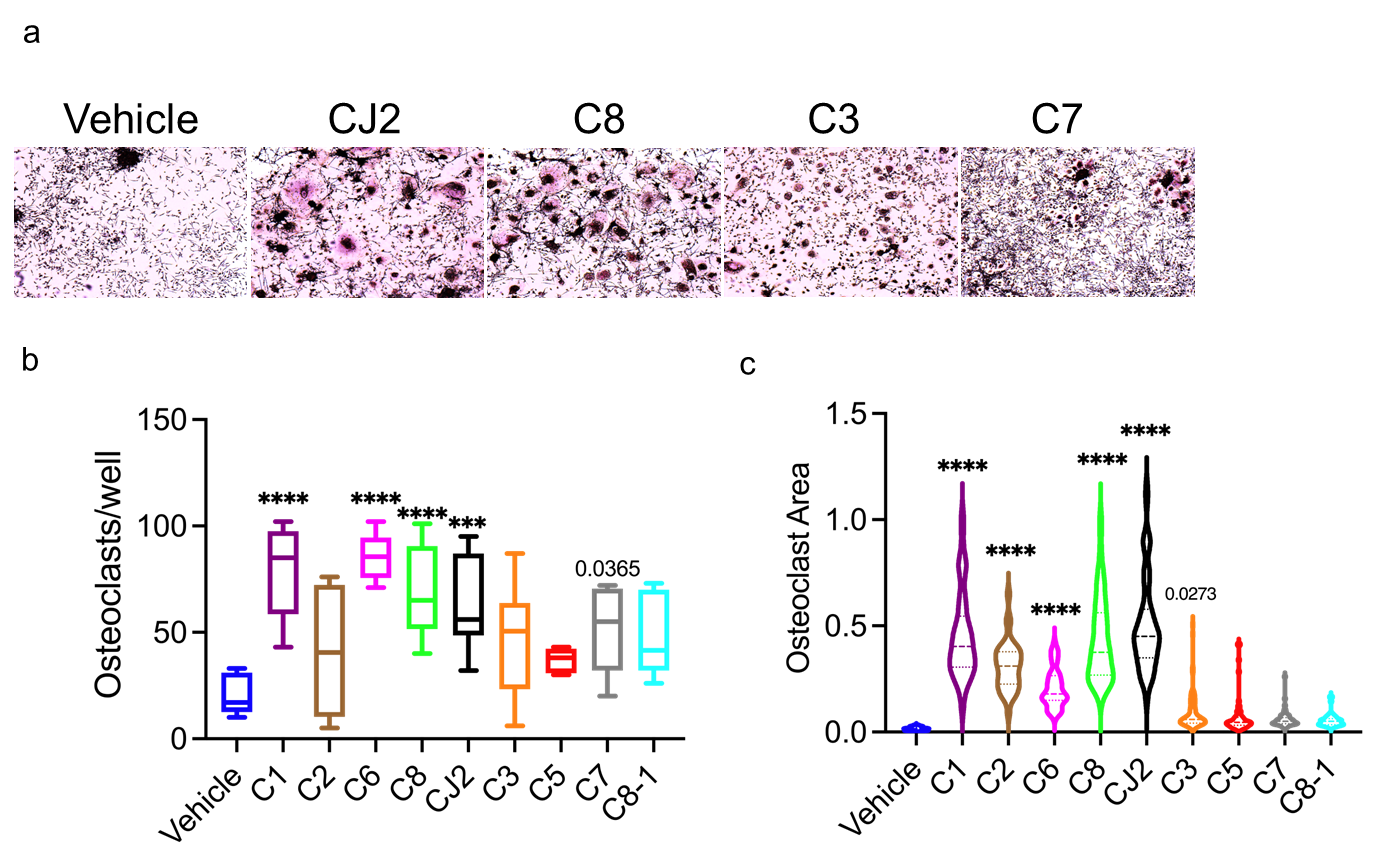


Supplementary Figure 1. Supernatants from HTLV/T cell lines variably affect differentiation of human PBMC to osteoclasts. a) Total human PBMC were treated with 10% HTLV/T supernatant. The cells were cultured for 5-6 days, fixed and subjected to TRAP staining. b) Multinucleated cells with >3 nuclei were counted. c) Average area of the osteoclasts was measured using Image J. Data from at least two independent experiments. n=6-9. Scale bar 200 μm, One-way ANOVA: * p<0.05; *** p<0.001; ****p<0.0001.


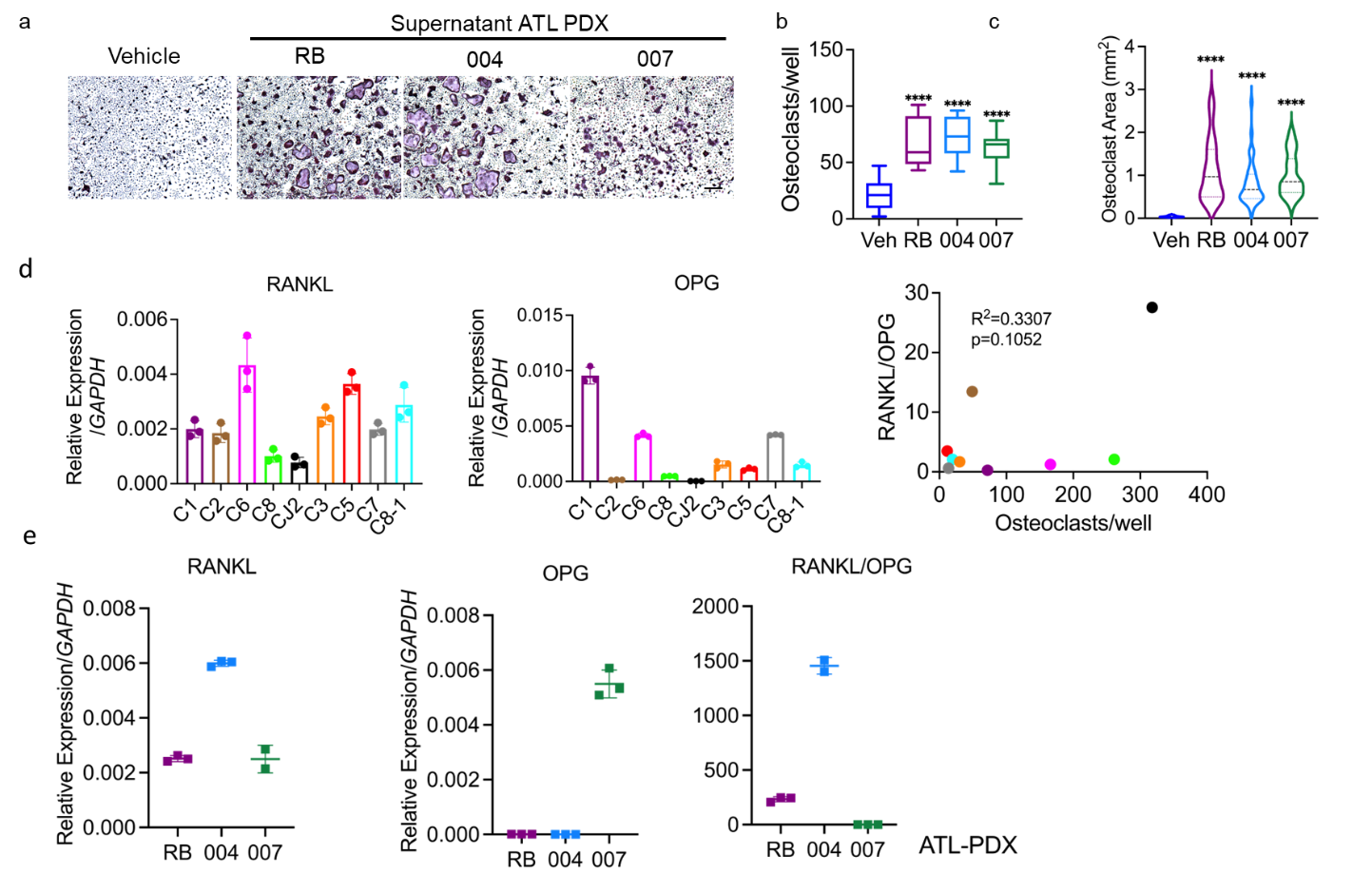


Supplementary Figure 2. Stimulation of osteoclastogenesis does not correlate with RANKL, OPG expression levels or RANKL/OPG ratio. a-c) Mouse bone marrow macropahges (mBMM) were treated with 10% supernatant from ATL-PDX RB, 004, ad 007. Scale bar: 500 μm, TRAP positive multinuclear cells (MNCs) were counted as osteoclasts b) Osteoclast number. c) Osteoclast area. 3 independent experiments, n=9. One-way ANOVA,  _****;_p<0.0001. d) Cellular mRNA expression level of RANKL and OPG by HTLV/T cell lines and their correlation to OC numbers in mBMM cultures. Pearson correlation, two-tailed, 95% CI. d) Cellular mRNA expression level of RANKL and OPG by ATL-PDX. RB,004 and 007 are ATL PDX.


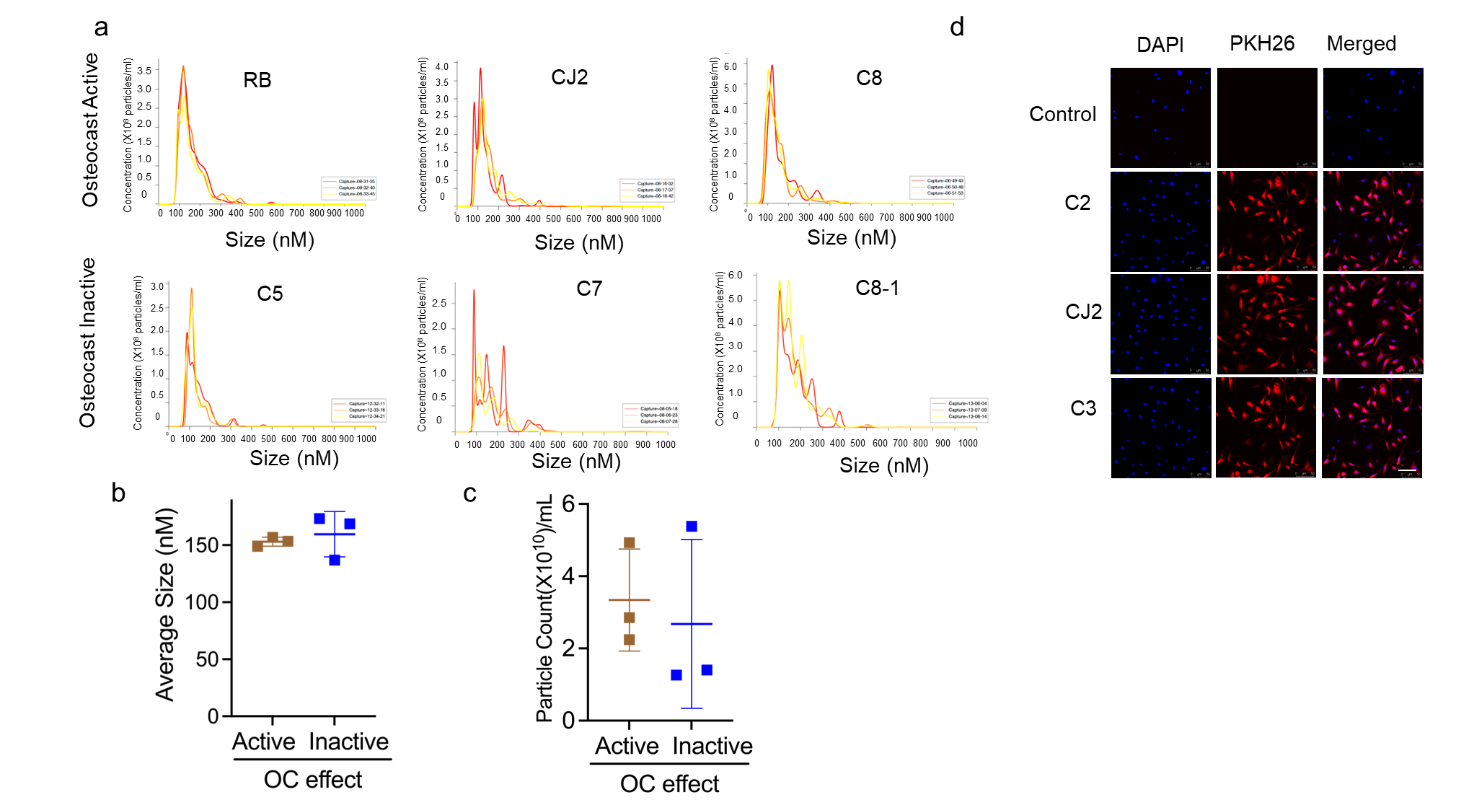


Supplementary Figure 3. sEV characterization. a) Representative images of sEV from cell lines with osteoclast active (RB,CJ2 and C8) and osteoclast inactive effect (C5, C7 and C8-1) analyzed by Nano Sight 300 for their size distribution and particle counts. Colors represent separate runs from the same sample. b) Average size and c) concentration of sEV particles from a. d) sEV from osteoclast active (C2,CJ2) and inactive (C3) HTLV/T were labelled with fluorescent dye PKH 26 and subsequently cultured with mBMM for 4 h. The mBMM were then fixed, stained with DAPI and imaged. Scale bar 100 μm.


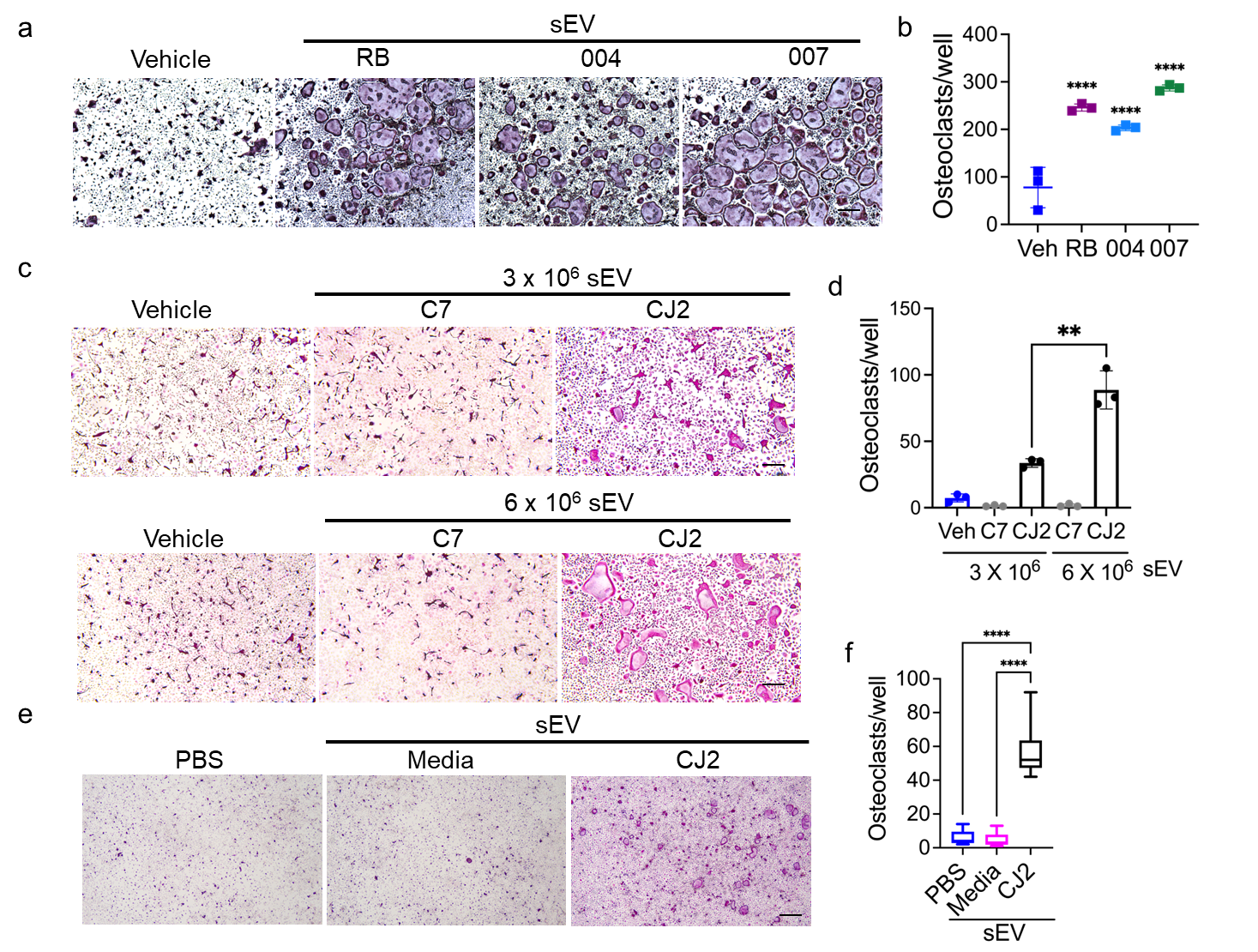


Supplementary Figure 4: sEV effect on osteoclastogenesis. a-b) sEV were precipitated from ATL-PDX culture supernatant and added to mBMM culture. TRAP positive multinuclear cells (MNCs) were counted as osteoclasts. Data represents technical replicates. c-d) sEV were precipitated from culture supernatant and added to mBMM culture at two different doses. TRAP positive multinuclear cells (MNCs) were counted as osteoclasts. Data represents technical replicates. e-f) sEV were precipitated from similar volume of unconditioned media (RPMI+ FBS) and culture supernatant from HTLV/T CJ2 and added to mBMM culture, compared to PBS. TRAP positive MNCs were counted as osteoclasts. Data represents 2 biological replicates. Scale bar 500 μm, One-way ANOVA, _**;_p<0.01 _****;_p<0.0001.


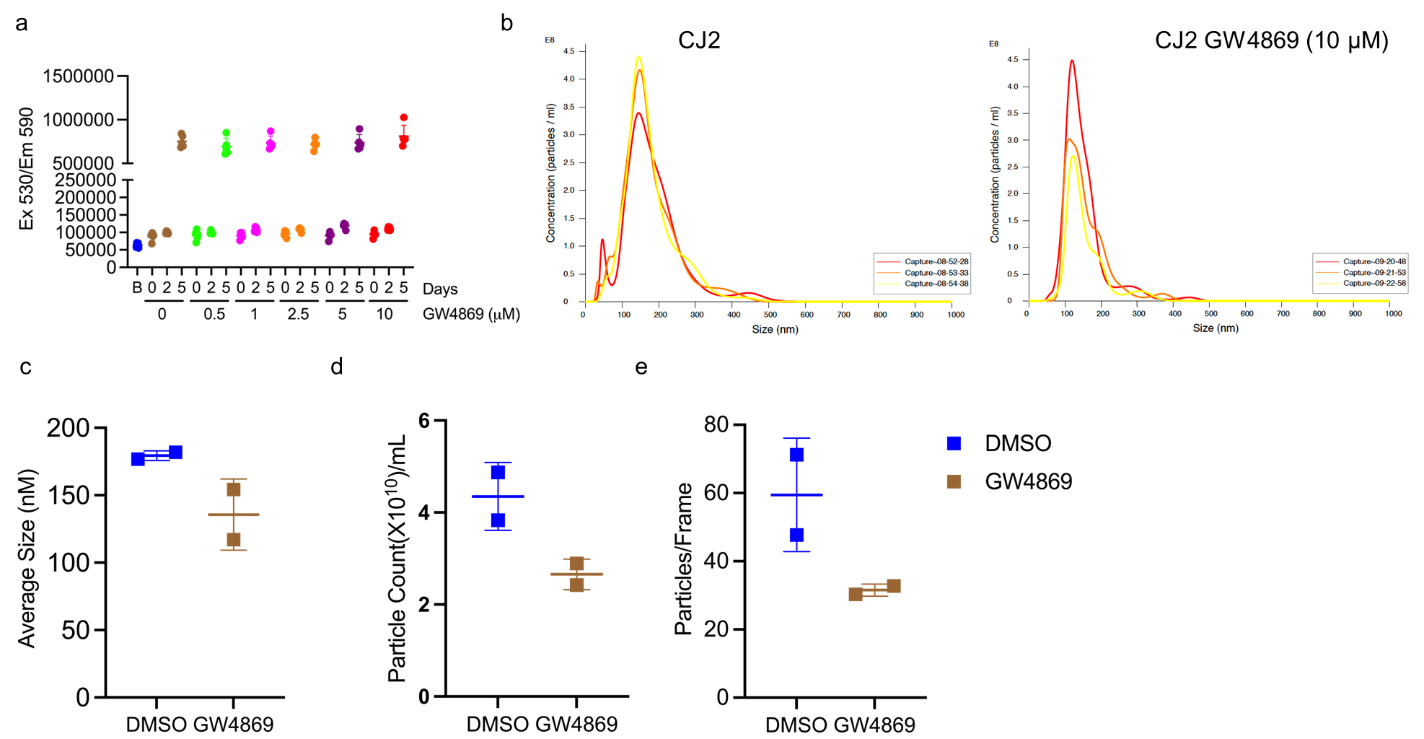


Supplementary Figure 5: Effect of GW4869 on cell viability and EV generation. a) HLTV/T CJ2 was treated with increasing amounts of GW4869 in DMSO as indicated and cell viability was assessed using MTT assay after 2 and 5d (B-Blank). b) Effect of GW4869 on EV generation by HTLV/T. HTLV/T (CJ2 and C8) sEV from either DMSO or GW4869 (10 μM) treated were analyzed by Nanosight 300 c) Average size, d) Particle counts/mL and e) Particles/Frame.


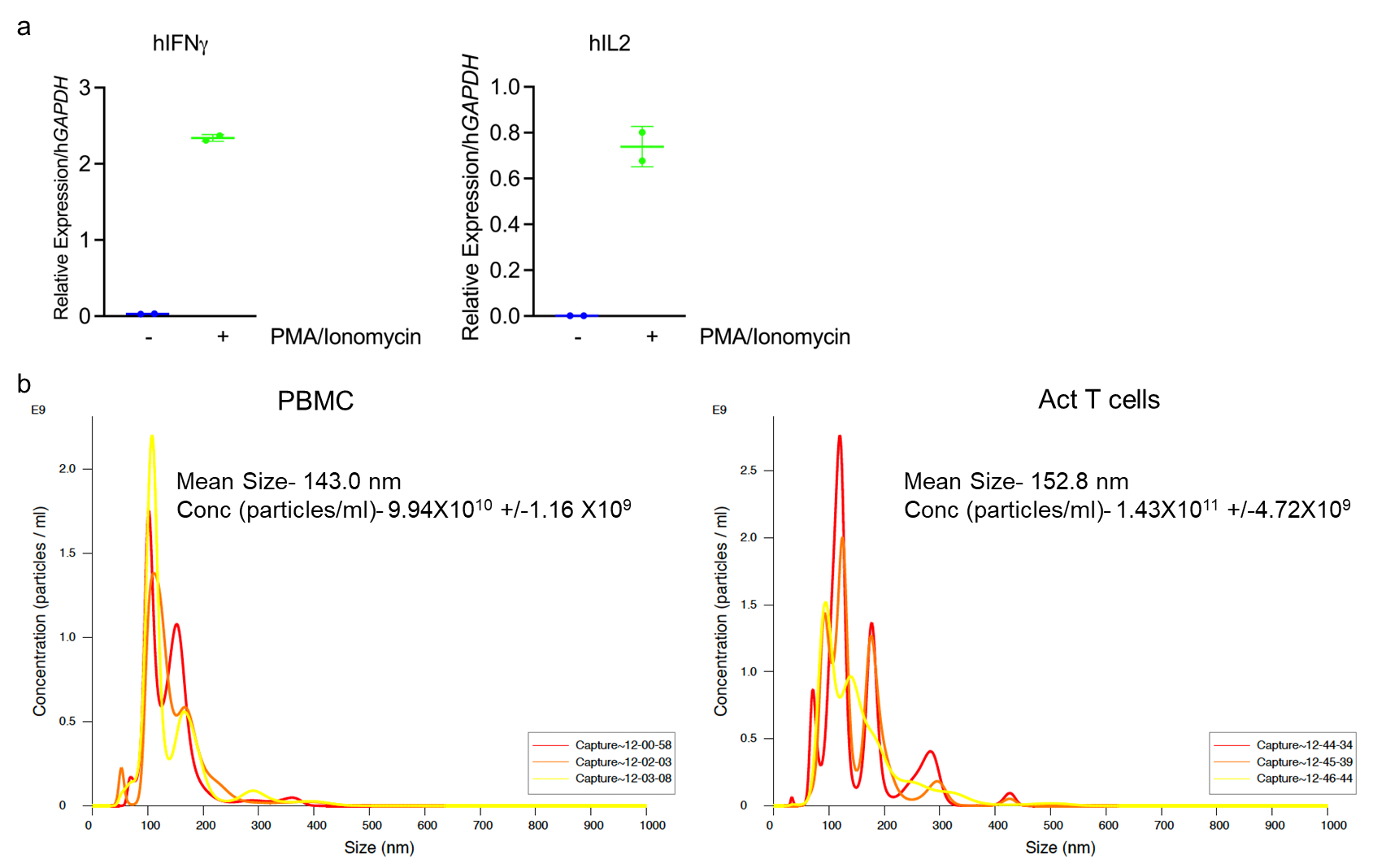


Supplementary Figure 6: T cell activation in the absence of infection did not affect EV production. PBMCs were cultured in hIL-2, in the absence or presence of PMA/Ionomycin for 48h. a) T cell activation by PMA/ Ionomycin was confirmed by examining hIFN𝛾 and IL2 mRNA expression. Technical replicates. b) sEV were precipitated from culture supernatant from same set of cells and analyzed using Nanosight 300. Technical replicates.


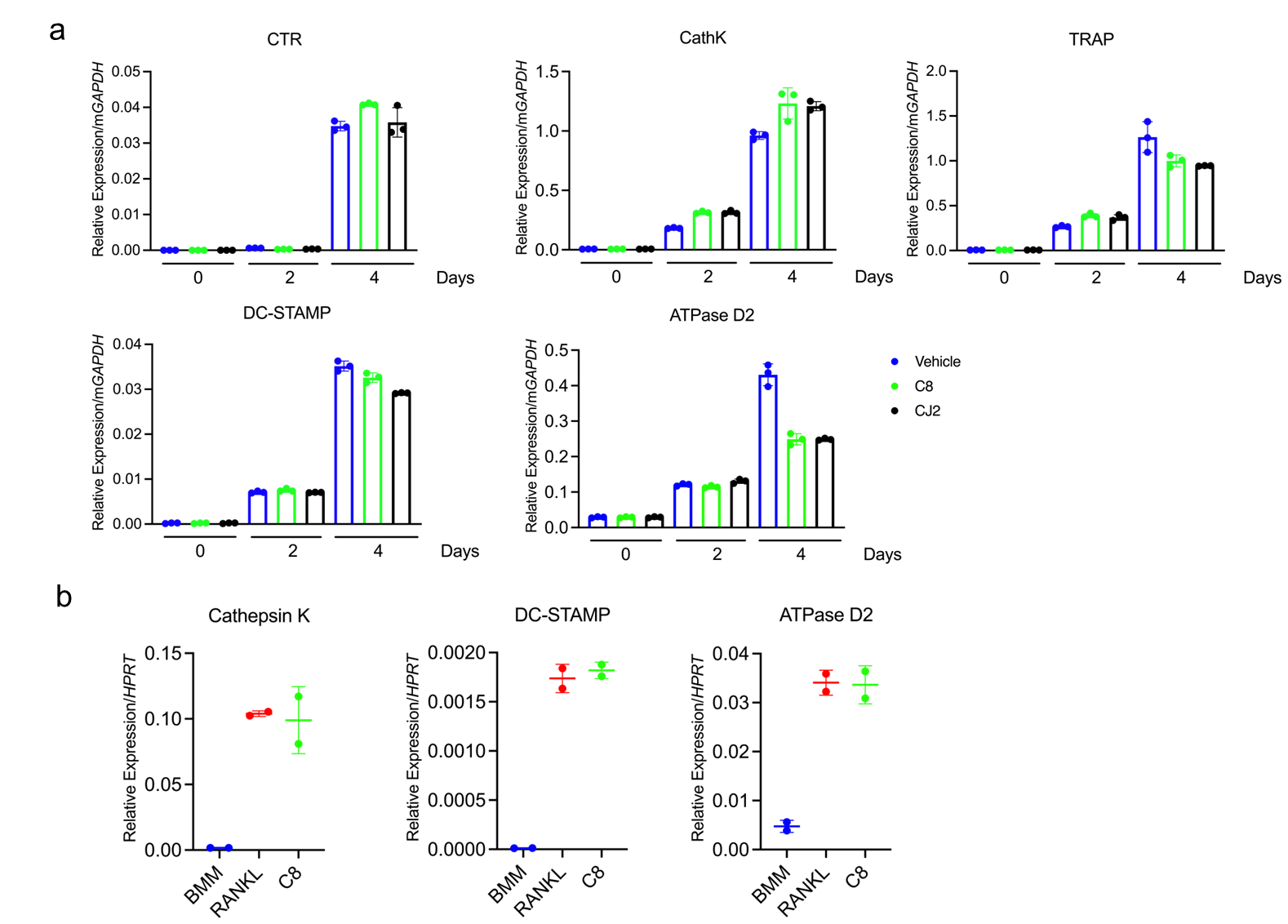


Supplementary Figure 7. sEV have no effect on expression of osteoclast marker genes. a) Cellular expression level of osteoclast differentiation genes during osteoclastogenic culture of mBMM with HTLV/T supernatant from the indicated HTLV/T lines. Data represents technical replicates. b) Pre-osteoclasts were generated from mBMM with a 2 d RANKL treatment, and sEV were added for an additional 24h prior to assessment of expression level of genes associated with osteoclast fusion. Data representative of at least 2 independent experiments.


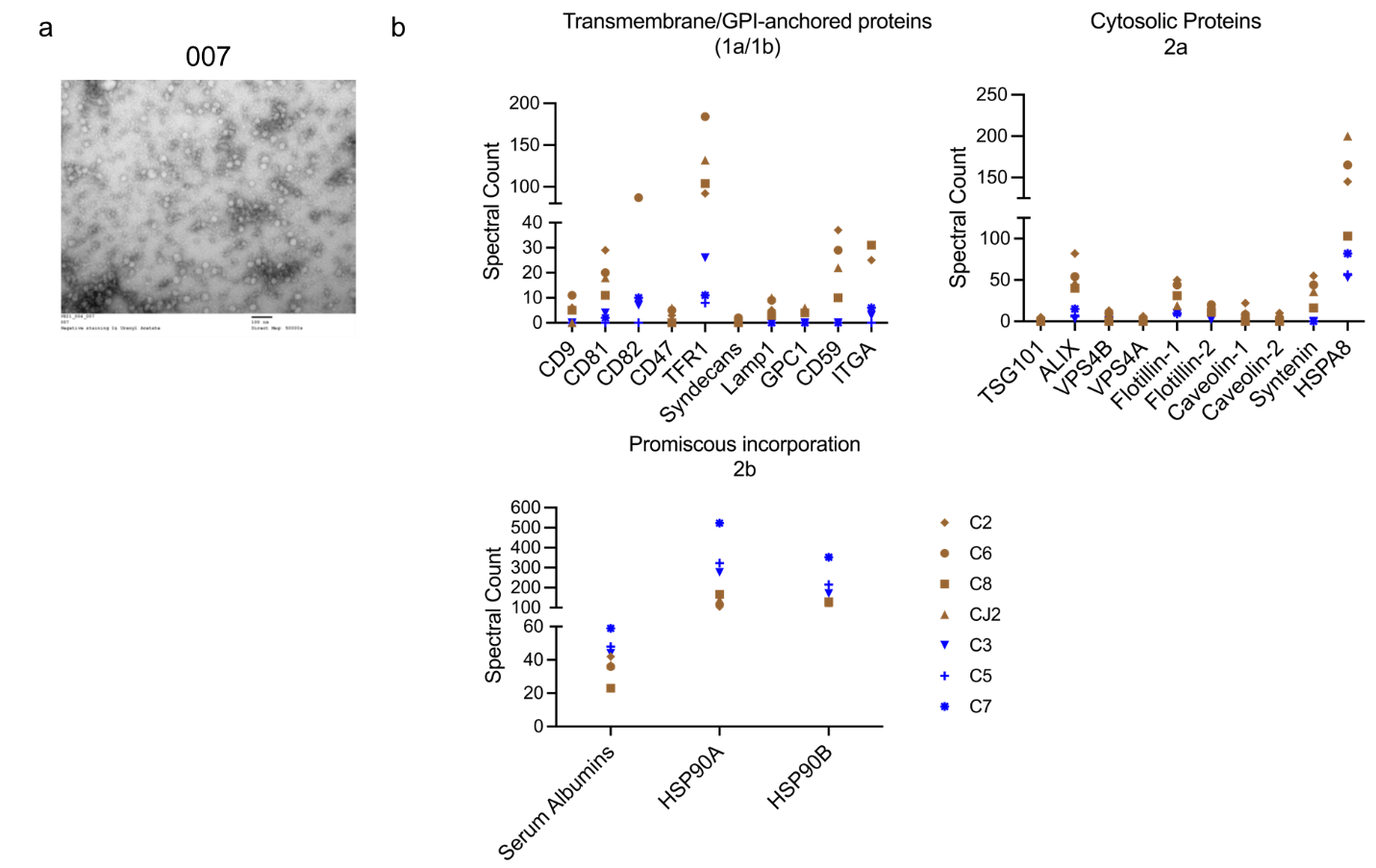


Supplementary Figure 8: sEV from HTLV/T cells do not contain virus, but do conform to MISEV guidelines for exosomes. a) sEV were negatively stained with uranyl acetate and examined by TEM. b) LC-MS/MS analysis was performed on sEV preparations (Brown- Osteoclast active, Blue-Osteoclast inactive). Exosomal marker distribution (spectral count) is presented, as defined by MISEV guidelines^31^.


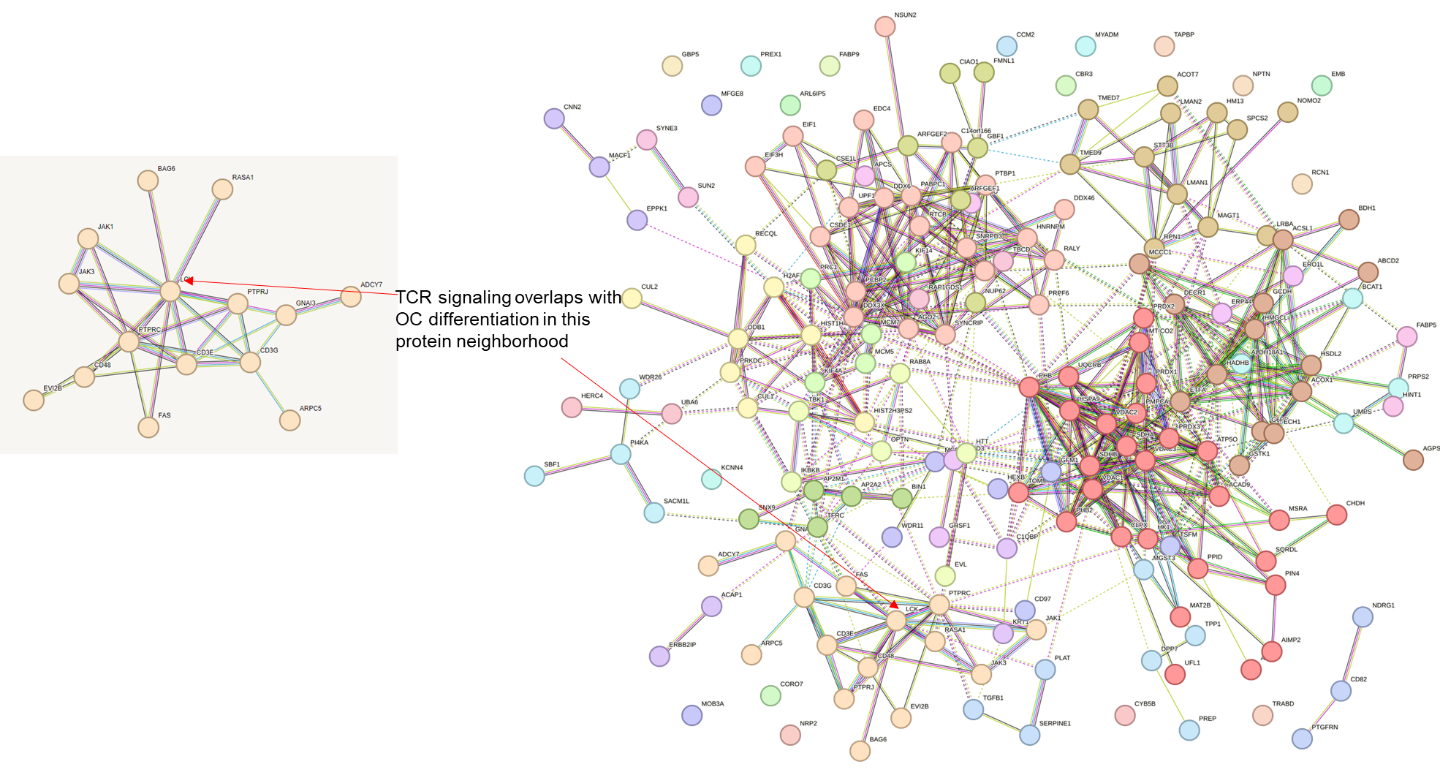


Supplementary Figure 9: Interaction of TCR signaling with osteoclast differentiation demonstrated by STRING analysis of sEV proteomics.

Supplementary Figure 10: Western blots for Env gp46, Flotillin-1, Calreticulin and Thrombospondin 1. sEV from HTLV/T CJ2 and C7 were isolated and expression of HTLV-1 envelope protein Env/gp46 (a), flotillin1 (b), Calreticulin (c) and thrombospondin 1 (TSP1) (d) were evaluated by immunoblot. Equal amounts of total protein for lysate and sEV were loaded. Full uncut blots with ladders shown. Arrow indicates expected band size.

Supplementary Table 1: List of Primers Used

| **For Genomic DNA qPCR** | Forward 5’-3’ | Reverse 5’-3’ |
| --- | --- | --- |
| Human *IL6* | ATTGGGAGCCCACACTCGAA | ATCACCTAGTCCACGCCCAA |
| Mouse *B2m* | ATGCTGAGGACCTTGTGAGC | GGAAGGCAGTAGGGAGAGGA |
| **For RT-qPCR** |  |  |
| Mouse TRAP (*Acp5*) | CGACCATTGTTAGCCACATACG | TCGTCCTGAAGATACTGCAGGTT |
| Mouse Cathepsin K (*Ctsk*) | ATATGTGGGCCAGGATGAAAGTT | TCGTTCCCCACAGGAATCTCT |
| Mouse Calcitonin receptor (*CALCR*) | GCAACCGAACCTGGTCCAACTAT | AAGCAGCAATCGACAAGGAGTGA |
| Mouse Dcstamp (*DCSTAMP*) | GACCTTGGGCACCAGTATTT | CAAAGCAACAGACTCCCAAA |
| Mouse v-ATPase (*ATP6v0d2*) | AGTGCAGTGTGAGACCTTGG | TCTGCAGAGCTTCTTCCTCA |
| Human RANKL (*TNFSF11*) | GGCCACAGCGCTTCTCAG | GAGTGACTTTATGGGAACCCGAT |
| Human IL2 | AACCTCAACTCCTGCCACAA | GCATCCTGGTGAGTTTGGGA |
| Human IFNγ | TGCAATCTGAGCCAGTGCTT | GCACCAGGCATGAAATCTCC |
| Human OPG (*TNFRSF11B*) | AGTCCGTGAAGCAGGAGTG | CCATCTGGACATTTTTTGCAAA |
| Human *GAPDH* | TGTGATGGGTGTGAACCACGAGAA | GAGCCCTTCCACAATGCCAAAGTT |
| Mouse *Hprt1* | CCTAAGATGATCGCAAGTTG | CCACAGGGACTAGAACACCTGCTAA |
| Mouse *GAPDH* | AGGTCGGTGTGAACGGATTTG | TGTAGACCATGTAGTTGAGGTCA |

Supplementary Table 2: Comparison of two different sEV isolation methods

| Protein | V96-Peptide based method | Precipitation Method  (Thermo scientific Kit) |
| --- | --- | --- |
| Actin, cytoplasmic | 929 | 722 |
| Fascin | 896 | 738 |
| Talin-1 | 259 | 540 |
| 60 kDa heat shock protein | 352 | 256 |
| Myosin-9 | 288 | 546 |
| Actin, alpha cardiac muscle 1 | 322 | 694 |
| Annexin A6 | 282 | 348 |
| Glyceraldehyde-3-phosphate dehydrogenase | 219 | 330 |
| Tubulin alpha-1B chain | 250 | 170 |
| Tubulin alpha-4A chain | 241 | 320 |
| Plectin | 238 | 300 |
| Vimentin | 221 | 211 |
| Alpha-enolase | 262 | 144 |
| Testican-2 | 247 | 380 |
| Filamin-A | 234 | 33 |
| Unconventional myosin-Ig | 206 | 290 |
| Histone H2B type 1-K | 208 | 211 |
| Tubulin beta chain | 198 | 39 |
| Moesin | 199 | 266 |
| Elongation factor 1-alpha 1 | 229 | 143 |
| Pyruvate kinase | 167 | 196 |
| ATP synthase subunit beta, mitochondrial | 158 | 225 |
| Tubulin beta-4B chain | 151 | 195 |
| Elongation factor 1-alpha 2 | 169 | 239 |
| Voltage-dependent anion-selective channel protein 1 | 144 | 147 |
| Histone H2B type 1-J | 126 | 345 |
| Heat shock protein HSP 90-beta | 152 | 31 |
| Neuroblast differentiation-associated protein | 145 | 643 |
| Basement membrane-specific heparan sulfate proteoglycan core protein | 147 | 169 |
| Cytoplasmic dynein 1 heavy chain 1 | 125 | 10 |
| Heat shock protein HSP 90-alpha | 157 | 164 |
| Tubulin beta-2B chain | 154 | 616 |
| Myosin regulatory light chain 12B | 0 | 208 |
| Agrin | 129 | 52 |
| Tubulin alpha-1C chain | 119 | 12 |
| Ras GTPase-activating-like protein | 122 | 117 |
| HLA class I histocompatibility antigen, A-68 alpha chain | 161 | 122 |
| Alpha-actinin-4 | 118 | 134 |
| Myosin-10 | 139 | 124 |
| Tubulin beta-4A chain | 140 | 204 |
| Receptor-type tyrosine-protein phosphatase C | 134 | 200 |
| Keratin, type I cytoskeletal 10 | 153 | 129 |
| Calreticulin | 106 | 86 |
| Leucine-rich PPR motif-containing protein, mitochondrial | 113 | 185 |
| Elongation factor 2 | 106 | 120 |
| Clathrin heavy chain 1 | 131 | 181 |
| Hypoxia up-regulated protein 1 | 125 | 153 |
| Stress-70 protein, mitochondrial | 86 | 94 |
| Hemoglobin subunit alpha | 96 | 229 |
| Periostin | 153 | 50 |
| Protein disulfide-isomerase A4 | 102 | 41 |
| Heat shock cognate 71 kDa protein | 84 | 110 |
| ATP synthase subunit alpha, mitochondrial | 97 | 199 |
| Sodium/potassium-transporting ATPase subunit alpha-1 | 73 | 114 |
| Keratin, type II cytoskeletal 1 | 107 | 105 |
| E3 ubiquitin-protein ligase | 103 | 117 |
| Integrin beta-2 | 101 | 130 |
| Fatty acid synthase | 125 | 74 |
| Plexin-B2 | 102 | 90 |
| E3 ubiquitin-protein ligase RNF213 | 99 | 69 |
| Endoplasmin | 139 | 110 |
| Ezrin | 117 | 252 |
| Keratin, type II cytoskeletal 2 epidermal | 131 | 111 |
| 14-3-3 protein theta | 88 | 96 |
| Prolow-density lipoprotein receptor-related protein 1 | 103 | 56 |
| Gelsolin GSN | 94 | 62 |
| HLA class I histocompatibility antigen, | 82 | 18 |
| Endoplasmic reticulum chaperone BiP | 94 | 108 |
| Raftlin | 91 | 210 |
| Voltage-dependent anion-selective channel protein 2 | 83 | 50 |
| Keratin, type I cytoskeletal 9 | 62 | 174 |
| Alpha-actinin-1 | 73 | 130 |
| Aconitate hydratase, mitochondrial | 86 | 109 |
| Tubulin beta-3 chain | 67 | 40 |
| Transitional endoplasmic reticulum ATPase | 93 | 181 |
| Vitamin D-binding protein | 82 | 69 |
| DNA-dependent protein kinase catalytic subunit | 111 | 32 |
| Guanine nucleotide-binding protein G(i) subunit alpha-2 | 85 | 57 |
| Histone H4 | 98 | 193 |
| Trifunctional enzyme subunit alpha, mitochondrial | 84 | 41 |
| Annexin A5 | 71 | 51 |
| HLA class I histocompatibility antigen, B-73 alpha chain | 72 | 73 |
| Galectin-1 | 91 | 127 |
| Fructose-bisphosphate aldolase A | 94 | 102 |
| Serum albumin | 75 | 116 |
| Kinesin-like protein KIF23 | 112 | 45 |
| Plastin-2 | 91 | 43 |
| Phosphoglycerate kinase 1 | 90 | 134 |
| Nucleophosmin | 80 | 108 |
| 2-oxoglutarate dehydrogenase, mitochondrial | 108 | 91 |
| Beta-actin-like protein 2 | 64 | 53 |
| Neutral alpha-glucosidase AB | 91 | 72 |
| 3-ketoacyl-CoA thiolase, mitochondrial | 72 | 66 |
| Cofilin-1 | 46 | 50 |
| T-complex protein 1 subunit epsilon | 68 | 196 |
| Heat shock 70 kDa protein 1A | 64 | 71 |
| Putative HLA class I histocompatibility antigen, alpha chain H | 75 | 114 |
| Histone H2A type 1-B/E | 71 | 53 |
| Thrombospondin-1 | 77 | 51 |
| Beta-enolase | 78 | 301 |
| Tubulin beta-6 chain T | 94 | 114 |
| Guanine nucleotide-binding protein G(I)/G(S)/G(T) subunit beta-1 | 78 | 61 |
| Cytochrome b-c1 complex subunit 2, mitochondrial | 87 | 113 |
| Ras GTPase-activating-like protein | 40 | 38 |
| Calnexin | 85 | 45 |
| Nucleobindin-1 | 59 | 55 |
| Protein disulfide-isomerase | 83 | 12 |
| 14-3-3 protein epsilon | 61 | 108 |
| Na(+)/H(+) exchange regulatory cofactor | 72 | 76 |
| Guanine nucleotide-binding protein | 78 | 17 |
| Lymphocyte-specific protein 1 | 75 | 75 |
| SUN domain-containing protein 2 | 70 | 37 |
| Sodium/potassium-transporting ATPase subunit alpha-3 | 65 | 28 |
| Elongation factor Tu, mitochondrial | 62 | 55 |
| WD repeat-containing protein 1 | 48 | 57 |
| Chloride intracellular channel protein 1 | 65 | 107 |
| Radixin | 91 | 61 |
| Protein disulfide-isomerase A3 | 63 | 75 |
| Acetyl-CoA acetyltransferase, mitochondrial | 47 | 83 |
| Very long-chain specific acyl-CoA dehydrogenase, mitochondrial | 62 | 51 |
| 14-3-3 protein beta/alpha | 45 | 44 |
| Major vault protein | 82 | 56 |
| 60S ribosomal protein L3 | 47 | 76 |
| Brain acid soluble protein 1 | 59 | 56 |
| Microtubule-actin cross-linking factor 1, isoforms 1/2/3/5 | 52 | 39 |
| Prothrombin | 56 | 33 |
| Integrin alpha-L | 56 | 119 |
| Delta(3,5)-Delta(2,4)-dienoyl-CoA isomerase, mitochondrial | 68 | 71 |
| Tumor necrosis factor alpha-induced protein 3 | 46 | 78 |
| eIF-2-alpha kinase activator GCN1 | 45 | 52 |
| Rac GTPase-activating protein 1 | 47 | 39 |
| Serine hydroxymethyltransferase, mitochondrial | 71 | 19 |
| Creatine kinase B-type | 37 | 50 |
| Transferrin receptor protein 1 | 41 | 26 |
| Guanine nucleotide-binding protein G(i) subunit alpha | 46 | 214 |
| Ubiquitin-like modifier-activating enzyme 1 | 66 | 118 |
| U5 small nuclear ribonucleoprotein 200 kDa helicase | 51 | 42 |
| E3 ubiquitin-protein ligase UBR4 | 46 | 77 |
| Adenylyl cyclase-associated protein 1 | 51 | 62 |
| Cullin-associated NEDD8-dissociated protein 1 | 50 | 54 |
| Glutaminase kidney isoform, mitochondrial | 50 | 54 |
| Calmodulin-1 | 32 | 54 |
| Neutral amino acid transporter A | 57 | 26 |
| Pre-mRNA-processing-splicing factor 8 | 60 | 18 |
| T-cell surface glycoprotein CD5 | 53 | 61 |
| Signal transducer and activator of transcription 5A | 53 | 59 |
| UDP-glucose:glycoprotein glucosyltransferase 1 | 54 | 41 |
| Fibronectin | 38 | 36 |
| Dedicator of cytokinesis protein 2 | 64 | 78 |

| HTLV/T | MIR155 | MIR21 | MIR92A1 | MIR22 | MIR146A | MIR191 | MIR221 | MIR92A2 |
| --- | --- | --- | --- | --- | --- | --- | --- | --- |
| C2 | 288,852 | 90,496 | 79,019 | 47,044 | 37,814 | 29,901 | 29,270 | 17,297 |
| C6 | 331,967 | 55,952 | 57,119 | 29,165 | 20,443 | 31,452 | 25,449 | 8,926 |
| C8 | 142,384 | 167,010 | 113,455 | 30,296 | 40,678 | 24,471 | 21,145 | 25,197 |
| CJ2 | 257,441 | 61,269 | 125,482 | 15,470 | 18,872 | 34,009 | 22,747 | 16,089 |

Supplementary Table 3: micro-RNA sequencing of sEV from osteoclast active HTLV/T. Counts per million (CPM) reads shown.

Supplementary Methods

Western Blotting

Intact cells or sEV precipitates (washed twice in PBS) were lysed using radioimmune precipitation assay buffer (20 mM Tris (pH 7.5), 150 mM NaCl, 1 mM EDTA, 1 mM EGTA, 1% Triton X-100, 2.5 mM sodium pyrophosphate, 1 mM β-glycerophosphate, 1 mM Na3VO4, and 1 mM NaF) with 1:50 HALT protease and phosphatase inhibitor (Thermo Fisher Scientific, Waltham, MA; 1861280). Lysates were vortexed and centrifuged at 700 × g. Protein was quantified with a BCA assay (Bio-Rad), and 20 μg was loaded to a 10% SDS-PAGE gel. Gels were run at 80 V for 2 h and transferred to nitrocellulose by wet transfer at 100 V for 60 min. Blocking was done in TBS and 0.1% Tween 20 with 5% milk. Membranes were probed with primary antibodies overnight at 4 °C, using the following reagents at indicated dilutions:

Flotillin-1: clone D2V7J, rabbit mAb #18634 Cell Signaling, 1:1000

Thrombospondin-1: clone D7E5F, rabbit mAb #37879, Cell Signaling 1:1000

Env/gp 46: mouse mAb, clone #68/4.11.21, # 0801085, Zeptometrix, 1:500

Calreticulin: clone D3E6 rabbit mAb #12238 Cell Signaling, 1:1000

Membranes were washed thrice with PBST and probed with secondary antibodies from LI-COR (Donkey anti-rabbit/IRDye or anti-mouse IRDye 680RD) at 1:10,000 dilution. Membranes were washed three times and scanned using scanned using LI-COR Odyssey Imager (LI-COR Biosciences, Lincoln, NE, USA) at low resolution using Licor software (RRID: SCR_014579).

sEV isolation using Vn peptides

The conditioned cell culture media was cleared by centrifugation at 17,000g and the supernatant was transferred to a new tube. EV precipitation was initiated by addition of 50 µL Vn96 peptide stock (New England Peptide, Gardner,US) (prepared per manufacturer instructions) followed by mixing via inversion, and incubation overnight at 4 ^o^C with end-over-end rotation. The incubated samples were again centrifuged at 17,000g at 4 ^o^C for 15 minutes using a bench-top microcentrifuge. sEV precipitates were visible at the bottom of tubes. sEV samples were washed three times with phosphate buffered saline (PBS). The precipitated Vn96-EV complexes were processed for proteomics analysis.

Proteomics:

Sample Preparation

Exosomes isolated from human cell culture samples were solubilized in 35 µl of SDS buffer (4% (wt/vol)), 100 mM Tris-HCl pH 8.0) with sonication in a water bath sonicator (VWR, 150D) at room temperature for 10 min at power level 9. Protein disulfide bonds were reduced using 100 mM dithiothreitol (DTT; Pierce, cat. no. 20291) with heating to 95 ºC for 10 min. Samples were digested as previously described^1^. Reduced samples were mixed with 600 µl 100 mM Tris-HCL buffer, pH 8.5 containing 8 M urea (Sigma, cat no. U4884-500g) (UA buffer), transferred to the top of a 30,000 molecular weight cut-off filter (Millipore, part# MRCF0R030) and spun in a microcentrifuge (Eppendorf) at 10,000 rcf for 10 min. An additional 300 µl of UA buffer was added and the filter was spun at 10,000 rcf for 10 min in a microcentrifuge. The flow through was discarded and the proteins were alkylated using 100 µl of 50 mM iodoacetamide (IAM, Pierce, Cat. No. A39271) in UA buffer. IAM in UA buffer was added to the top chamber of the filtration unit. The samples were gyrated at 550 rpm using a Thermomixer (Eppendorf) at room temperature for 30 min in the dark. The filter was spun at 10,000rcf for 10 min and the flow through discarded. Unreacted IAM was washed through the filter with two additions of 200 µl of UA buffer, and centrifugation at 10,000 rcf for 10 min after each buffer addition. The UA buffer was exchanged with digestion buffer (DB), 50 mM ammonium bicarbonate buffer, pH 8. Two sequential additions of DB (200 µl) with centrifugation after each addition to the top chamber was performed. The filters were transferred to a new collection tube and 100 µl DB containing 1 mAU of LysC (Wako Chemicals, cat. no. 129-02541) was added and samples were incubated at 37 °C for 2 h. Trypsin (1 µg) (Promega, Cat. No. V5113) was added and samples were incubated overnight at 37 °C. The filters were spun at 10,000 rcf for 15 min to collect the peptides in the flow through. The filter was washed with 50 µL 100mM ammonium bicarbonate buffer and the wash was collected with the peptides. Peptides were acidified with trifluoroacetic acid (TFA; Sigma, cat. no. 91707) to a final concentration of 1% (vol/vol) and were desalted using two micro-tips (porous graphite carbon, BIOMETNT3CAR) (Glygen) on a Beckman robot (Biomek NX)^2^. The peptides were eluted with 60% (vol/vol) acetonitrile (MeCN; J.T. Baker, cat. no. 9829-03) in 0.1% (vol/vol) TFA and dried in a Speed-Vac (Thermo Scientific, Model No. Savant DNA 120 concentrator). Samples were dissolved in 20 µl of 1% (vol/vol) MeCN in water. An aliquot (10%) was removed for quantification using the Pierce Quantitative Fluorometric Peptide Assay kit (Thermo Scientific, Cat. No. 23290). The remaining peptides were transferred to autosampler vials (Sun-Sri, Cat. No. 200046), dried, and stored at -80 °C.

UPLC-timsTOF-MS.

The peptides were analyzed using trapped ion mobility time-of-flight mass spectrometry^3^. Peptides were separated using a nano-ELUTE chromatograph (Bruker Daltonics. Bremen, Germany) interfaced to a timsTOF Pro mass spectrometer (Bruker Daltonics) with a modified nano-electrospray source (CaptiveSpray, Bruker Daltonics). The mass spectrometer was operated in PASEF mode^3^. The samples in 2 µl of 1% (vol/vol) formic acid (FA; Sigma-Aldrich, cat. no. 56302) were injected onto a 75 µm i.d. × 25 cm Aurora Series column with a CSI emitter (Ionopticks). The column temperature was set to 50 °C. The column was equilibrated using constant pressure (800 bar) with 8 column volumes of solvent A (0.1% (vol/vol) FA. Sample loading was performed at constant pressure (800 bar) at a volume of 1 sample pick-up volume plus 2 µl. The peptides were eluted using one column separation mode with a flow rate of 300 nl/min and using solvents A (0.1% (vol/vol) FA) and B (0.1% (vol/vol) FA/MeCN): solvent A containing 2% B increased to 17% B over 60 min, to 25% B over 30 min, to 37% B over 10 min, to 80% B over 10 min and constant 80% B for 10 min. The MS1 and MS2 spectra were recorded from m/z 100 to 1700.

The collision energy was ramped stepwise as a function of increasing ion mobility: 52 eV for 0–19% of the ramp time; 47 eV from 19–38%; 42 eV from 38–57%; 37 eV from 57–76%; and 32 eV for the remainder. The TIMS elution voltage was calibrated linearly using the Agilent ESI-L Tuning Mix (m/z 622, 922, 1222).

MS data analysis.

The MS2 spectra from peptides with +2, +3 and +4 charge states were analyzed using Mascot software^4^ (Matrix Science, London, UK; version 2.8.0.1). Mascot was set up to search against a UniProt reference databases of human proteins (20,512 entries, downloaded February 2021) and common contaminant proteins (cRAP, v1.0 Jan. 2012; 116 entries), assuming the digestion enzyme was trypsin with a maximum of 4 missed cleavages allowed. The searches were performed with a fragment ion mass tolerance of 20 ppm and a parent ion tolerance of 20 ppm. Carbamidomethylation of cysteine was specified in Mascot as a fixed modification. Deamidation of asparagine, deamidation of glutamine, pyro-glutamate formation from n-terminal glutamine, acetylation of protein N-terminus and oxidation of methionine were specified as variable modifications. Peptide spectrum matches (PSM) were filtered at 1% false-discovery rate (FDR) by searching against a reversed database and the ascribed peptide and protein identities were accepted.

The processing, quality assurance and analysis of LC-MS data were performed with proteoQ (version 1.5.0.0, https://github.com/qzhang503/proteoQ), software developed with the tidyverse approach^5,6^ with open-source software for statistical computing and graphics^7,8^. The precursor intensities were converted to logarithmic ratios (base 2), relative to the average precursor intensity across all samples. Within each sample, Dixon’s outlier removals were carried out recursively for peptides with greater than two identifying PSM’s. The median of the ratios of PSM that could be assigned to the same peptide was first taken to represent the ratios of the incumbent peptide. The median of the ratios of peptides was then taken to represent the ratios of the inferred protein. To align protein ratios across samples, likelihood functions were first estimated for the log-ratios of proteins using finite mixture modeling, assuming two-component Gaussian mixtures^9^. The ratio distributions were then aligned so that the maximum likelihood of log-ratios was centered at zero for each sample. Scaling normalization was performed to standardize the log-ratios of proteins across all samples. To reduce the influence of outliers from either log-ratios or reporter-ion intensities, the values between the 5th and 95th percentile of log-ratios and 5th and 95th percentile of intensity were used in the calculations of standard deviations.

Bioinformatic and statistical analysis.

Metric multidimensional scaling (MDS) and Principal component analysis (PCA) of protein log2-ratios was performed with the base R function stats:cmdscale and stats:prcomp, respectively. Heat-map visualization of protein log2-ratios was performed with heatmap^10^. Linear modelings were performed using the contrast fit approach in limma^11^, to assess the statistical significance in protein abundance differences between indicated groups of contrasts. Adjustments of p-values for multiple comparison were performed with Benjamini-Hochberg (BH) correction.

Proteomics method references

1. Wiśniewski JR, Zougman A, Nagaraj N, Mann M. Universal sample preparation method for proteome analysis. Nat Methods. 2009 May;6(5):359-62.
2. Chen ZW, Fuchs K, Sieghart W, Townsend RR, Evers AS. Deep amino acid sequencing of native brain GABAA receptors using high-resolution mass spectrometry. Mol Cell Proteomics. 2012 Jan;11(1):M111.011445.
3. Meier F, Brunner AD, Koch S, Koch H, Lubeck M, Krause M, Goedecke N, Decker J, Kosinski T, Park MA, Bache N, Hoerning O, Cox J, Räther O, Mann M. Online Parallel Accumulation-Serial Fragmentation (PASEF) with a Novel Trapped Ion Mobility Mass Spectrometer. Mol Cell Proteomics. 2018 Dec;17(12):2534-2545.
4. Perkins DN, Pappin DJ, Creasy DM, Cottrell JS. Probability-based protein identification by searching sequence databases using mass spectrometry data. Electrophoresis. 1999 Dec;20(18):3551-67.
5. Hadley Wickham. Advanced R, Second Edition (Chapman & Hall/CRC The R Series).
6. Hadley Wickham (2017). tidyverse: Easily Install and Load the 'Tidyverse'. R package version 1.2.1. <https://CRAN.R-project.org/package=tidyverse>.
7. R Core Team (2021). R: A language and environment for statistical computing. R Foundation for Statistical Computing, Vienna, Austria. URL <https://www.R-project.org/>).
8. RStudio (RStudio Team (2016). RStudio: Integrated Development for R. RStudio, Inc., Boston, MA URL <http://www.rstudio.com/>).
9. Benaglia, T., Chauveau, D., Hunter, D. R., & Young, D. S. (2009). mixtools: An R Package for Analyzing Mixture Models. Journal of Statistical Software, 32(6), 1–29.
10. Kolde, R. (2019). pheatmap: Pretty Heatmaps. R package version 1.0. 12.
11. Ritchie ME, Phipson B, Wu D, Hu Y, Law CW, Shi W, Smyth GK. limma powers differential expression analyses for RNA-sequencing and microarray studies. Nucleic Acids Res. 2015 Apr 20;43(7).
